# A Peer-Based Intervention to Increase HIV and Sexually Transmitted Infection Testing Among Latinx Immigrant Sexual Minority Men in the US Pacific Northwest: Pilot Randomized Controlled Trial Conducted During the COVID-19 Pandemic

**DOI:** 10.2196/45871

**Published:** 2023-07-12

**Authors:** Jane J Lee, Gabriel Robles, Christopher A Leyva Vera, E Roberto Orellana, Susan M Graham, Anh-Minh Nguyen, Yingying Wei, Abraham Hernandez Sanchez, Julia C Dombrowski, Jane M Simoni

**Affiliations:** 1 School of Social Work University of Washington Seattle, WA United States; 2 School of Social Work Rutgers University New Brunswick, NJ United States; 3 Department of Global Health University of Washington Seattle, WA United States; 4 Department of Medicine University of Washington Seattle, WA United States; 5 Department of Biostatistics University of Washington Seattle, WA United States; 6 HIV/STD Program Public Health – Seattle & King County Seattle, WA United States; 7 Department of Psychology University of Washington Seattle, WA United States

**Keywords:** HIV prevention, self-testing, sexually transmitted infections, preexposure prophylaxis, peers, Latinx immigrants

## Abstract

**Background:**

Hispanic and Latinx gay, bisexual, and other sexual minority men (SMM) are disproportionately affected by HIV in the United States. With the availability of self-testing services, HIV and sexually transmitted infection (STI) testing may be more accessible for Latinx immigrant SMM who face obstacles to obtaining HIV-related services. Combining the potential of self-testing kits and the influence of peer educators may present an opportunity to increase HIV and STI testing and preexposure prophylaxis (PrEP) uptake or linkage to HIV care among Latinx immigrant SMM.

**Objective:**

This study aimed to develop and pilot a peer intervention to distribute HIV and STI self-testing kits and provide peer counseling based on the information-motivation-behavioral skills model to increase PrEP uptake and HIV and STI testing among Latinx immigrant SMM. Our evaluation focused on determining the differences in HIV testing, STI testing, and PrEP uptake outcomes between the intervention and control groups.

**Methods:**

We conducted semistructured interviews with community stakeholders to elicit factors to consider for training and intervention. The interview findings informed the development of the intervention and peer training protocols. We piloted the intervention with Latinx immigrant SMM and randomly assigned participants to the intervention group, who received peer counseling and HIV and STI self-testing kits, or the control group, who only received peer counseling. We administered baseline, 1-week, 6-week, and 12-week follow-up surveys to assess behaviors related to HIV testing, STI testing, and PrEP uptake. Owing to the COVID-19 pandemic, the intervention components were delivered via web-based modalities. Chi-square tests were performed to examine the associations between HIV testing, STI testing, and PrEP motivation and behaviors across the study arms (intervention vs control). We conducted Cramer V test to determine the strength of the association between study arm and each of the outcome variables. We also assessed the impact of the COVID-19 pandemic on participants.

**Results:**

Overall, 50 (intervention, n=30 and control, n=20) Latinx immigrant SMM participated in the program. Participants reported life disruptions owing to COVID-19, with 68% (34/50) reporting job loss after the declaration of the pandemic. After intervention participation, a higher proportion of participants in the intervention group reported having been tested for STIs (76% vs 36.8%; *P=.*01; Cramer V=0.394). Among the participants in the intervention group, 91% (21/23) reported being motivated to use PrEP compared with 59% (10/17) in the control group (*P*=.02; Cramer V=0.385).

**Conclusions:**

By facilitating access to HIV and STI testing through peer-delivered information, motivational support, and behavioral skills training as well as the provision of self-testing kits, our intervention demonstrated the potential to increase HIV prevention behaviors in Latinx immigrant SMM. Peer-based programs that offer self-testing and internet-based modes of accessing information may be a feasible strategy for reaching Latinx immigrant SMM.

**Trial Registration:**

ClinicalTrials.gov NCT03922126; https://clinicaltrials.gov/ct2/show/NCT03922126

## Introduction

HIV disproportionately affects Hispanic and Latinx gay, bisexual, and other sexual minority men (SMM) in the United States [1]. Although Latinx SMM accounted for nearly one-fourth of all new HIV diagnoses in 2019, one in 5 Latinx SMM with HIV was unaware of their HIV status [1]. In King County, Washington, one of the priority jurisdictions that have accounted for a disproportionate burden of all new HIV diagnoses in the United States [2], Latinx SMM have an estimated HIV prevalence rate of 13.7% [3]. Notably, Latinx people with HIV in King County, Washington, are more likely to be diagnosed late or experience an AIDS diagnosis within 1 year of an HIV diagnosis [3], highlighting the importance of ensuring access to HIV prevention and care services for this population. Screening for HIV and other sexually transmitted infections (STIs) is a key strategy for HIV prevention and is an important step in accessing antiretroviral therapy (ART) for people with HIV and, for those who are HIV-negative, in the uptake of preexposure prophylaxis (PrEP), a highly effective intervention to reduce HIV acquisition risk, provided adherence is high [4].

However, Latinx SMM face multiple structural and social challenges in accessing HIV prevention and care services, including stigma, language barriers, discrimination, and socioeconomic factors [5,6]. Specifically, Latinx SMM may avoid seeking HIV prevention and care services because of negative experiences in health care and social. The inability to adequately communicate with providers or pay for services may further preclude Latinx SMM from HIV testing and ART or PrEP use [6]. These barriers may render HIV prevention and care services inaccessible or unacceptable for Latinx SMM and may require multiple strategies to increase service use in this community.

Prior research on HIV prevention and care in racial and ethnic minority and sexual minority communities has demonstrated that peers or individuals who share characteristics or experiences with the population of interest may increase the acceptability of HIV prevention and care interventions and influence behaviors such as HIV testing [7,8]. By providing tailored information or enhancing client motivation in one’s preferred language, peers may be more likely than other professionals to influence behavior change and reduce HIV transmission risk among fellow group members [9,10]. Furthermore, greater trust of peers can facilitate more effective and transparent interactions among SMM to contribute to improved health [11], suggesting that peer interventions have the potential to address multiple barriers to HIV prevention among Latinx SMM [10].

Another strategy that may reduce the challenges to HIV prevention is the availability of HIV and STI self-testing services. Currently, at-home tests are available for HIV, as are STI self-collection kits that can be submitted to a laboratory by mail [12]. The ability to self-collect specimens and receive results through phone, mail, or email can eliminate obstacles related to receiving a test at a clinic or other public location [12,13]. As Latinx SMM who are foreign born (herein referred to as Latinx immigrant SMM) may have concerns about privacy or worry about interactions with providers in clinical settings, HIV and STI self-testing may present an opportunity to access services that they might otherwise be unable or unwilling to seek.

Given the potential of peer-based interventions and self-testing kits in addressing specific barriers to HIV prevention and care, we combined peer counseling and HIV and STI self-testing services to develop a peer-based HIV prevention intervention for Latinx immigrant SMM. The intervention was informed by the information-motivation-behavior skills model of health behavior change [14,15], which has extensive empirical support for the development of behavioral interventions in the context of HIV prevention with key populations at risk for HIV and among people with HIV taking ART [15,16]. Peers provided counseling that included information about HIV and STI testing and PrEP, basic motivational interviewing for HIV prevention including PrEP among those testing negative, and the enhancement of behavioral skills to support engagement in HIV prevention services. The intervention also involved peer-supported delivery of HIV and STI self-testing kits for at-home specimen collection and submission; the interpretation of results once available; and linkage to treatment, as indicated. We sought peers to address the cultural barriers, language issues, and stigma that pose challenges to HIV prevention and care among Latinx immigrant SMM. The use of self-testing kits was intended to facilitate the accessibility of HIV and STI testing for this population. Taken together, the combination of peer counseling and HIV and STI self-testing has the potential to overcome multiple structural-, social-, and individual-level barriers to HIV prevention and care.

Notably, we launched intervention implementation at the start of the COVID-19 pandemic, which prompted shifts in study activities and the intervention protocol. Given the social distancing requirements and stay-at-home orders, we adapted all activities to be conducted remotely through web-based and phone modalities to avoid any in-person contact. The ramifications of the COVID-19 pandemic presented disruptions to all facets of life, potentially limiting access to HIV and STI prevention efforts. The delivery of our intervention during the COVID-19 pandemic (2020-2021) highlighted the challenges and opportunities of using peers and HIV and STI self-testing services for HIV and STI prevention and care among Latinx immigrant SMM.

In this paper, we report on the development and pilot-testing of a peer-based HIV and STI testing intervention. First, we describe how the intervention was developed and refined through qualitative interviews with community stakeholders. We then present the procedures and results of our pilot randomized controlled trial of the peer-based HIV and STI testing intervention. The pilot trial examined the impact of the COVID-19 pandemic on study participants and differences in self-reports of HIV testing, STI testing, and PrEP uptake outcomes between the intervention and control group participants.

## Methods

### Intervention Development

#### In-Depth Interviews

To elicit important factors to consider for training peers to deliver both education on HIV prevention and care and the HIV and STI self-testing intervention to Latinx immigrant SMM, we conducted semistructured in-depth interviews with community stakeholders (n=15) from diverse sectors, including community organizations (6/15, 40%), research (3/15, 20%), health care (4/15, 27%), and public health (2/15, 13%). Our objective in these interviews was to obtain perspectives from key stakeholders who are knowledgeable about HIV prevention in diverse Latinx populations to assess how best to work with peers and use HIV and STI self-testing as part of the intervention.

Interviews were facilitated by the study principal investigator (PI) and conducted over the phone or in-person based on the participant’s availability and preference. As the purpose of the qualitative interviews was to inform processes for our intervention, we used rapid qualitative analysis to summarize key points and themes from the data [17,18]. Specifically, we used tables and spreadsheets to analyze the data and deductively identify themes that focused on specific barriers to HIV and STI testing and PrEP use in the Latinx immigrant SMM community and ways in which the planned intervention could help overcome these barriers [19]. The PI and 2 additional members of the research team discussed the final data tables for revision and agreement. The tables resulted in focused codes or themes that answered our overarching question of how to address barriers to HIV prevention through our intervention. Given that this qualitative analysis was central to the development of our intervention and informed our approach to piloting the intervention, we present the results of the qualitative interviews as part of the methods of our main study.

In-depth interviews revealed themes related to (1) HIV and STI testing knowledge, (2) PrEP knowledge, (3) access to HIV prevention and care services, (4) the acceptability and feasibility of HIV and STI self-testing, and (5) the role of peers in supporting the intervention. The interviews helped inform intervention development, as presented in Table 1. For example, stakeholders noted that cost and financial concerns are major barriers to accessing HIV prevention and care services, especially for men without access to medical insurance. Hence, we ensured that participation in the peer and HIV and STI testing intervention would be free of cost to the participants. In addition, stakeholders believed that familiarity with HIV testing and self-collection of STI specimens may be low among Latinx immigrant SMM. Thus, we integrated education on the skills needed to appropriately self-collect specimens for testing.

Overall, the community stakeholder interviews validated the need for peer-based strategies for reaching Latinx immigrant SMM for HIV prevention. Several stakeholders explained how peers play critical roles in clinical and hospital settings to ensure that patients can make their appointments and navigate systems to access care and medication. They noted that this demonstrated the utility of peers in supporting Latinx immigrant SMM communities for facilitating HIV testing and PrEP uptake. Stakeholders also provided input on the types of HIV and STI tests to use and how to ensure appropriate follow-up and linkage to care.

Although our intervention presented an opportunity to offer a new way to access HIV and STI screening services, we wanted to ensure that peers delivering the intervention had the training and supervision needed to provide adequate support, information, and instructions on how to use the self-testing service. Thus, peer training, as described in the “Peer Recruitment and Training” section, involved role-plays and extensive practice in conducting self-testing kits to ensure that peers were prepared to troubleshoot potential problems. We also wanted to support peers by providing participants with explanations of the potential advantages and disadvantages of using such services. Specifically, peers were equipped with reasons why Latinx immigrant SMM may or may not be interested in HIV prevention and were provided with potential responses to concerns or questions about self-testing.

Our qualitative findings from stakeholders on recruitment strategies, procedures, and the availability of HIV and STI self-collection kits and clinical supports for diagnosis and treatment supported the study team in designing intervention materials, developing recruitment strategies for peers and study participants, and developing and finalizing the protocol for a randomized trial to pilot-test the intervention.

**Table 1:** Community stakeholder interview (n=15) themes and implications for the peer-based HIV prevention intervention for Latinx immigrant sexual minority men (SMM).

Interview themes and findings		Intervention and study implications
**HIV and STI^a^ testing knowledge**		
	Most Latinx immigrants have basic knowledge about HIV and AIDS and other STIs and are familiar with testing for HIV and STIs. The duration of time residing in the United States and country of origin may be linked to Latinx SMM’s knowledge about HIV and STIs and their understanding of how to get tested for HIV. Knowledge about self-testing services may be limited among Latinx immigrant SMM.	Take into account limited familiarity of how to access HIV testing services in the US context. Support participants in understanding local context and systems. Ask about participants’ knowledge of and experience with HIV and STI testing in their countries of origin to determine whether additional education is needed. Provide clear instructions and support for participants in using self-testing services for HIV and STIs.
**PrEP^b^ knowledge**		
	There is growing knowledge among Latinx immigrants about PrEP (what it is, how you use it, and where you can get it). There may be misinformation in the Latinx immigrant SMM community about what PrEP is and who should use it. Latinx immigrant SMM may be interested in PrEP but be unaware of the steps to accessing the medication. They may be unsure about the pros and cons about using PrEP. Latinx immigrant SMM can feel comfortable with using PrEP with the right information and guidance.	PrEP education must be a key component of the intervention. Ask the participant to explain their understanding of PrEP and its use. Discuss and address (if possible) concerns or misunderstandings about PrEP use.
**Access to HIV prevention services**		
	Very few culturally sensitive and accessible HIV prevention services in certain counties outside of the Seattle area. Many Latinx immigrants travel to Seattle to access HIV and other health services. Immigrant status and lack of health insurance are major factors that can impede care; although there are clinics that offer services to individuals who are undocumented and uninsured, they may be few and far between. Privacy concerns—some individuals may not want to go to clinics for HIV and STI services because they fear that they may encounter people that they know there. The “free” clinics are often overwhelmed, making wait times longer. Health insurance and documentation status are barriers to care. Language of providers and fear of discriminatory interactions with providers are barriers in the community—there are not enough providers who are from the Latinx community and speak Spanish.	Ensuring privacy and confidentiality is critical—conduct the intervention and meet with the participant in a location that is private. Offer neutral meeting spaces if the participant does not want to meet at their home. Peers who work with participants will be part of the community and understand the culture and language needs of the participants. Discussion about stigma (individual and community level) can be integrated into discussions to further motivate and empower participants to engage in HIV prevention. Peers can focus on targeting the intervention to participants in areas where services are less concentrated and identify potential participants who may be unable to obtain services during “regular business hours.” Highlight that involvement in the program is free.
**Role of peers**		
	Latinx SMM would benefit from peer support for accessing and using HIV prevention services. Peer navigators are key in helping many patients make their appointments and take their medications. Latinx immigrant SMM will likely be receptive to peer support for HIV and STI self-testing and PrEP use. Characteristics of peers that are important include language, expertise, and personability.	Empower peers and ensure that they clearly understand their role in the intervention. Ensure that peers can communicate with participants in their preferred language. Recruit a diverse cohort of peers who can address diverse cultural needs in the Latinx immigrant community.

^a^STI: sexually transmitted infection.

^b^PrEP: preexposure prophylaxis.

#### Intervention Components

The peer-based HIV and STI self-testing intervention was named *Listos* by community members during intervention development, which translates to “ready” or “smart.” The multiple meanings of the word were meant to highlight that Latinx immigrant SMM were not only ready for HIV prevention but were also smart for using HIV prevention services.

The intervention involved 2 main components. The first was peer counseling, which involved peers providing information about HIV and STI testing and PrEP, motivating participants to engage in HIV and STI testing and use PrEP, and reinforcing behavioral skills for HIV and STI testing and PrEP use. Peers also used empowerment strategies to help participants overcome the challenges in HIV prevention. The peer counseling session was conducted during the initial meeting between peers and participants after the baseline assessment. The intervention was considered “peer based,” given that it was delivered by peers. The second component was HIV and STI self-testing kits that were provided to the participants after the initial peer counseling session. On the basis of guidance from stakeholders, the intervention group received both intervention components, whereas the control group only received peer counseling.

For peer counseling, 3 separate modules were developed for peers to cover the participants during the initial individual session. The first involved providing relevant information about HIV and STI testing and PrEP, including what each entailed and their roles in preventing HIV transmission. The second involved a discussion about the reasons for testing for HIV and STI and using PrEP. Peers were to discuss the benefits of these HIV prevention behaviors and why an individual would get tested for HIV and STIs and use PrEP. Peers also discussed why HIV testing is key to linkage to ART initiation for undiagnosed people with HIV. The third module focused on relaying tailored information about how one would go about getting tested for HIV and STIs or using PrEP and reinforcing behavioral skills by providing step-by-step guidance on making appointments for HIV and STI testing and getting a prescription for PrEP. Overall, peers were supported as experts in the community to be able to motivate and empower others to engage in HIV prevention behaviors. Peers checked in with participants before each follow-up assessment and provided additional counseling as needed, which was guided by the modules.

The second component of the intervention involved HIV and STI self-testing kits that were provided to the participants after the initial peer counseling session. We used HIV and STI self-testing kits from Molecular Testing Labs that required self-collection of specimens and return mail submission for processing. The kits involved urine collection and blood spot collection using a lancet. Peers helped participants complete the self-collection kits by guiding them through the process and explaining how to use each of the items in the kit. Peers assisted the participants at each step and provided an overview of the instructions. The participants returned specimens via mail in postage-prepaid envelopes. Peers informed the participants of this step and reminded them to return the specimens. Molecular Testing Labs tested the specimens for HIV, *Chlamydia trachomatis, Neisseria gonorrhea, Trichomonas vaginalis*, hepatitis C antibody, herpes simplex virus type 2 (HSV-2), and syphilis. After returning the kits through mail, participants received their results via email within 7 days. Peers supported the participants in the interpretation of the test results once they were delivered to the participants. Peers connected participants who required additional follow-up care based on the test results to a local clinic, organization, or provider based on the participant’s preference and availability.

### Pilot Randomized Controlled Trial

#### Peer Recruitment and Training

Peers for the project met the following characteristics, which were also required of the study participants: (1) identify as Hispanic or Latinx, (2) immigrant or foreign born, (3) aged at least 18 years, (4) assigned male at birth, and (5) report sex with men. HIV status was not an exclusion criterion for participation as a peer. Peers were recruited through referrals from community-based organizations and linkages from prior research studies. Peers were interviewed and selected based on their interest in helping the community, experience with and understanding of HIV and STI prevention, and aptitude for supporting others to engage in HIV prevention behaviors. Peers were considered part of the study team and were compensated as consultants for their work in the study. Five peers were recruited to implement the intervention. The average age of the peers was 34 (SD 7.0; range 24-41) years, and the average duration of time residing in the United States was 10 (SD 5.5; range 2-16) years. A total of 4 peers were born in Mexico, and 1 of the peers was born in Honduras.

Before peer training in March 2020, the World Health Organization declared COVID-19 a pandemic, and US states began to shut down to prevent the spread of COVID-19 [20]. To protect the safety of peers and to follow local public health guidelines, all research activities were temporarily paused. We subsequently adapted the peer training and intervention procedures to be conducted remotely using internet and phone technologies. The 3-day peer training was conducted over Zoom (Zoom Video Communications), and all relevant materials and training supplies were mailed or emailed to peers.

The training sought to support peers in delivering the intervention with fidelity. At the end of the training, the facilitator conducted mock sessions with each of the peers to ensure that they were knowledgeable about all aspects of the intervention and study protocol, covered each of the content areas, provided correct and adequate information, and used appropriate techniques to motivate and empower participants. The PI maintained regular contact and follow-up with peers to support fidelity in the delivery of the intervention. Specifically, the PI supervised all peers, which included regular check-ins with peers over Zoom and email communication regarding the study procedures.

#### Recruitment

Participants were recruited from February 2021 to December 2021 through the peers’ social networks, word-of-mouth, and referrals. Peers also used social media outreach strategies, such as messaging individuals in Facebook groups, to recruit potential participants. This recruitment strategy was a shift from initially planned efforts to recruit individuals using street intercept methods at community events, bars, and other social gathering locations. The eligibility criteria included (1) identify as Hispanic or Latinx, (2) immigrant or foreign born, (3) aged at least 18 years, (4) assigned male at birth, (5) report sex with men, and (6) and report unknown or HIV-negative status. We excluded participants who were known to be HIV positive and would therefore not be eligible for PrEP.

#### Study Procedures

Assessments were conducted via web-based surveys at baseline (prerandomization) and at 1, 6, and 12 weeks after the intervention. The surveys were self-administered using REDCap (Research Electronic Data Capture; Vanderbilt University) and were available in Spanish or English [21]. After the participants completed their baseline assessment, they were randomized to the intervention or control group using computer-generated simple randomization. Peers provided reminders about completing the surveys and offered support for survey completion. The survey assessments took approximately 30 to 60 minutes to complete.

Given the adaptation for the COVID-19 pandemic, peers communicated with participants via phone, email, SMS text message, and other internet-based communication methods (eg, Facetime and WhatsApp). After randomization, participants in both groups received their first individual counseling session with their peers, which lasted approximately 45 minutes. Participants in both arms also received follow-up sessions approximately 1 week before the 6- and 12-week surveys. Participants in both groups were able to access their peers via phone, email, or SMS text message throughout the course of the intervention. Only participants in the intervention group received HIV and STI testing kits, which were mailed to their homes after randomization. For these participants, kits were registered on their behalf by the study PI, and peers provided internet-based support for completing the kits as part of the initial peer counseling session. Specifically, peers walked participants through the completion of the self-testing kits via phone or video chat. Peers had sample kits to demonstrate and explain the testing procedures to the participants.

### Ethics Approval, Informed Consent, and Participation

All study procedures were reviewed and approved by the Institutional Review Board at the University of Washington under FWA #00006878 (STUDY00007054). All participants provided informed consent before participating in the study. The participants received a US $35 electronic gift card incentive after completing each assessment. The study was granted a federal Certificate of Confidentiality, and the data collected were kept confidential.

### Measures

Surveys asked about participants’ age, education, annual household income, country of origin, and immigration status and whether participants had ever returned to their country of origin since they migrated to the United States. The participants also reported their sexual identity, religion, and whether they had medical insurance. Baseline surveys asked participants about their HIV testing behaviors (ever tested for HIV and time since the last HIV test) and the result of their last HIV test if they had ever tested for HIV. Baseline surveys also asked about their STI testing behaviors (whether they had ever tested for gonorrhea, chlamydia, syphilis, hepatitis B, hepatitis C, genital warts, and genital herpes) and the result of their last STI test if they had ever been tested for any STI. PrEP use behaviors were also assessed at baseline with questions that asked whether participants were aware of PrEP, whether they had talked with their health care provider about PrEP, whether they had used PrEP in the last 12 months, and whether they were currently taking PrEP. Added questions at baseline and follow-ups owing to the COVID-19 pandemic included the experience of COVID-19 symptoms, COVID-19 testing and test results, and job loss. The impact of COVID-19 on participants was assessed by a question that asked how much COVID-19 affected their day-to-day lives on a scale of 1=“not at all” to 5=“an extreme amount.” Distress related to COVID-19 was assessed by a question that asked, “In general, how much distress have you experienced in relation to COVID-19,” with response options ranging on a scale of 1=“no distress” to 10=“extreme distress.” Finally, participants indicated whether they were scared of being diagnosed with COVID-19 (yes, no, or do not know).

At each follow-up assessment, participants were asked about their HIV testing behaviors, STI testing behaviors, and PrEP use behaviors since the baseline assessment. Participants were also asked whether participating in the program motivated them to get tested for HIV or for STIs since the baseline assessment (yes or no). Finally, participants reported whether they had started taking PrEP since the completion of the baseline assessment and whether participation in the program motivated them to use PrEP.

For the intervention group participants who received HIV and STI self-testing kits, we received information from Molecular Testing Labs regarding whether they received completed testing kits. Participants who completed the testing kits received the results through email. The PI also sent these results to ensure that peers reviewed the results with participants and ensured that there was appropriate linkage to follow-up care. The location at which care was provided depended on the location of the participant and whether they had a preferred or primary care provider.

### Statistical Analyses

We calculated descriptive statistics for all variables, including those related to COVID-19 and tested for differences between the intervention and control groups at baseline using Fisher exact tests or *t* tests to ensure that the groups were appropriately randomized. To assess the differences in self-reported outcomes related to HIV testing, STI testing, and PrEP uptake between the intervention and control groups after program participation, we performed 2-sided *χ*^2^ tests at *P*=.05 significance level. Cramer V was used to measure the strength of the association between the intervention condition (*Listos* intervention or control) and each outcome variable. Cramer V values range from 0 to 1, with values closer to 1 indicating a stronger association. As this was a pilot study, the analyses were not powered to detect the intervention effects. Study analyses to assess preliminary intervention efficacy were based on the intent-to-treat sample, and all randomized participants were included in the analyses. Given the small sample sizes and incomplete follow-up data for all participants, we combined responses to all follow-up assessments to determine differences by the 12-week assessment point. Specifically, a last observation carried forward approach was used, where missing follow-up data were replaced by the participant’s previously observed value.

## Results

### Overview

**Figure 1:**
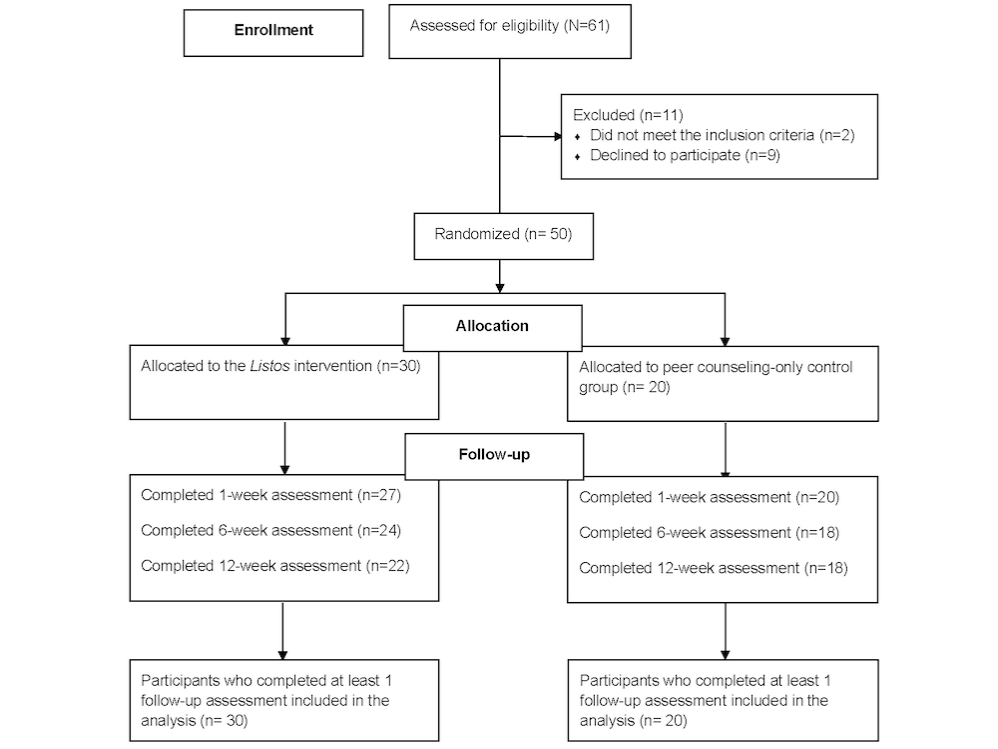
CONSORT (Consolidated Standards of Reporting Trials) participant flowchart.

We screened 61 individuals for eligibility, 59 (97%) of whom met the eligibility criteria. Of the 59 eligible participants, 50 (85%) agreed to participate. All participants who were eligible and interested in the study provided informed consent. At study completion, 73% (22/30) of the participants in the *Listos* intervention group and 90% (18/20) of those in the control group completed their 12-week assessment (Figure 1).

### Participant Characteristics

Table 2 presents the baseline demographic characteristics of participants in the intervention and control groups. Most participants (41/50, 82%) completed the survey in Spanish, and the overall mean age was 37.3 years. Overall, 62% (31/50) of participants reported being born in Mexico, and less than one-third (15/50, 30%) of the participants indicated having ever returned to their country of origin since migrating to the United States. Approximately a quarter (13/50, 26%) of the participants indicated that they were legal permanent residents, and another quarter (12/50, 24%) indicated that they were unauthorized immigrants. Moreover, 70% (35/50) of participants were identified as Christian or Catholic. Less than half (22/50, 44%) of participants had medical insurance. Sociodemographic characteristics did not differ significantly between the groups (all *P*>.05).

**Table 2:** Baseline demographic characteristics by intervention and control groups.

		Overall (n=50)	Intervention (n=30)	Control (n=20)	*P* value^a^	
Age (years), mean (SD)		37.3 (10.8)	34.9 (11.0)	41 (9.7)	.049^b^	
**Education, n (%)**						.96
	Less than high school	13 (26)	8 (27)	5 (25)		
	High school	9 (18)	6 (20)	3 (15)		
	Some college	10 (20)	6 (20)	4 (20)		
	College degree or higher	17 (36)	10 (33)	8 (40)		
**Annual household income (US $), n (%)**						.43
	<10,000	10 (20)	7 (23)	3 (15)		
	10,000-29,999	13 (26)	8 (27)	5 (25)		
	30,000-39,999	11 (22)	8 (27)	3 (15)		
	40,000-49,999	7 (14)	4 (13)	3 (15)		
	≥50,000	9 (18)	3 (10)	6 (30)		
**Immigration status, n (%)**						.08
	Legal permanent resident	13 (26)	4 (13)	9 (45)		
	Naturalized citizen	4 (8)	2 (6.7)	2 (10)		
	Unauthorized immigrant	12 (24)	10 (33)	2 (10)		
	Eligible immigrant or temporary resident	11 (22)	8 (27)	3 (15)		
	Other	9 (18)	6 (20)	3 (15)		
**Country of origin, n (%)**						.049
	Mexico	31 (62)	20 (67)	11 (55)		
	Colombia	7 (14)	1 (3)	6 (30)		
	El Salvador	3 (6)	2 (7)	1 (5)		
	Honduras	4 (8)	4 (13)	0 (0)		
	Other	5 (10)	3 (10)	2 (10)		
**Ever returned to the country of origin since migrating to the United States, n (%)**						.17
	Yes	15 (30)	7 (23)	8 (40)		
**Religion, n (%)**						.16
	Christian	13 (26)	11 (37)	2 (10)		
	Catholic	22 (44)	11 (37)	11 (55)		
	Agnostic	1 (2)	1 (3)	0 (0)		
	Spiritual but not religious	8 (16)	3 (10)	5 (25)		
	No religion or other	6 (12)	4 (13)	2 (10)		
**Sexual identity, n (%)**						.46
	Homosexual or gay	40 (80)	25 (83)	15 (75)		
	Bisexual	6 (12)	3 (10)	3 (15)		
	Heterosexual	1 (2)	1 (3)	0 (0)		
	Queer	1 (2)	0 (0)	1 (5)		
	Pansexual	1 (2)	0 (0)	1 (5)		
	Other	1 (2)	1 (3)	0 (0)		
Has medical insurance, n (%)		22 (44)	12 (40)	10 (50)	.77	

^a^Fisher exact test *P* values unless otherwise stated.

^b^*t* test *P* value.

### COVID-19–Related Characteristics

Table 3 presents the characteristics related to COVID-19 among the participants overall and by intervention and control groups. There were no differences between the arms. At baseline, approximately one-third (18/50, 36%) of participants reported having experienced COVID-19 symptoms, whereas the vast majority (43/50, 86%) of participants indicated having been tested for COVID-19. Overall, 28% (12/43) of participants who had tested for COVID-19 had received a positive test result, and more than two-thirds (34/50, 68%) of the participants had lost their jobs since the declaration of the COVID-19 pandemic. The average impact of COVID-19 on participants’ lives was 3.4 on a scale of 1 to 5, and the average level of distress experienced in relation to COVID-19 was 5.9 on a scale of 1 to 10. Approximately half (26/50, 52%) of the participants noted that they were scared of COVID-19.

**Table 3:** COVID-19–related characteristics by intervention and control groups.

	Overall (n=50)	Intervention (n=30)	Control (n=20)	*P* value^a^
Experienced COVID-19 symptoms, n (%)	18 (36)	13 (43)	5 (25)	.23
Tested for COVID-19, n (%)	43 (86)	26 (87)	17 (85)	.87
Received a positive COVID-19 test result^b^, n (%)	12^c^ (28)	8^d^ (31)	4^e^ (24)	.61
Lost job since declaration of COVID-19 pandemic, n (%)	34 (68)	21 (62)	13 (65)	.71
Impact of COVID-19, numeric score, mean (SD)	3.41 (1.19)	3.62 (1.15)	3.10 (1.21)	.13^f^
Distress related to COVID-19, numeric score, mean (SD)	5.92 (3.25)	6.37 (3.33)	5.25 (3.09)	.24^f^
Scared about COVID-19, n (%)	26 (52)	17 (57)	9 (45)	.42

^a^Fisher exact test *P* values, unless otherwise stated.

^b^Asked among those who indicated having been tested for COVID-19.

^c^n=43.

^d^n=26.

^e^n=17.

^f^*t* test *P* value.

### Intervention and Control Group Differences in HIV Testing, STI Testing, and PrEP Uptake

With respect to HIV testing results, the vast majority (48/50, 96%) of participants reported having ever being tested for HIV, and approximately one-third (18/50, 36%) of the participants reported having ever used PrEP at baseline. Differences in HIV testing, STI testing, and PrEP uptake outcomes between the intervention and control groups after participation in the program are presented in Table 4. Most participants indicated that the program motivated them to get tested for HIV and STIs (proportion in the intervention group: 23/25, 92% and 24/25, 96%, respectively; proportion in the control group: 12/18, 66.7% and 13/19, 68.4%, respectively; *P*<.05). The analysis revealed a significant association between assigned group and motivation to get tested for HIV (Cramer V=0.321; *P*=.04) and motivation to get tested for STIs (Cramer V=0.373; *P*=.01). A considerable proportion of all participants (30/44, 68.2%) reported testing for HIV after participating in the program. There were no differences in the reports of HIV testing after completion of the program between the intervention and control groups. A greater proportion of participants in the intervention group reported testing for STIs than those in the control group (19/25, 76% vs 7/19, 37%; *P=*.009; Cramer V=0.394). The overall proportion of participants who indicated that the program motivated them to use PrEP was 78% (31/40), with 91% (21/23) in the intervention group and 59% (10/17) in the control group (Cramer V=0.385; *P*=.02). Overall, of the 43 participants, 10 (23%) reported initiating PrEP after participating in the program (n=8, 80% in the intervention group and n=2, 20% in the control group).

Among the 30 *Listos* intervention group participants who received the HIV and STI self-testing kits, 20 (67%) completed kits that were received by the testing laboratory. Notably, only 19 intervention group participants self-reported having been tested for HIV after participating in the program (Table 4), which may have resulted from differences in the timing of assessment completion and completion of the testing kit. Five of these participants had positive results: 4 for HSV-2, 2 for syphilis, and 1 for HIV. For 3 participants, positive results for HSV-2 were new diagnoses based on self-reports. All 5 participants who received a positive test result received counseling from their peers and were linked to appropriate follow-up testing and care through local community–based service providers.

**Table 4:** Intervention and control group differences in HIV testing, sexually transmitted infection (STI) testing, and preexposure prophylaxis (PrEP) uptake outcomes^a,b^.

	Overall, n (%)		Intervention, n (%)		Control, n (%)			*χ* ^*2*^ *(df)*		*P* value^c^		V^d^
	n (%)	N	n (%)	N	n (%)	N						
Motivated to get tested for HIV	35 (81)	43	23 (92)	25	12 (67)	18	4.435 (1)		.04		0.321	
Tested for HIV	30 (68)	44	19 (76)	25	11 (58)	19	1.631 (1)		.20		0.193	
Motivated to get tested for STIs	37 (84)	44	24 (96)	25	13 (68)	19	6.138 (1)		.01		0.373	
Tested for STIs	26 (59)	44	19 (76)	25	7 (37)	19	6.848 (1)		.01		0.394	
Motivated to use PrEP	31 (78)	40	21 (91)	23	10 (59)	17	5.914 (1)		.02		0.385	
Started taking PrEP	10 (23)	43	8 (32)	25	2 (11)	18	2.559 (1)		.11		0.110	

^a^All outcomes were based on reports conducted after participating in the intervention or control groups. Responses to follow-up assessments (1, 6, and 12 weeks) were combined, and any “yes” response to each outcome measures was coded as “yes” for the question.

^b^Total sample sizes were <50 owing to missing data.

^c^Pearson *χ^2^* test.

^d^Cramer V.

## Discussion

### Principal Findings

This study demonstrated the feasibility of the *Listos* program, a peer-based HIV and STI intervention conducted remotely during the COVID-19 pandemic. The goal of the program was to promote HIV testing, STI testing, and PrEP use among Latinx immigrant SMM in Washington State. Our primary objective was to examine the differences in HIV testing, STI testing, and PrEP use behaviors between the intervention and control groups. In addition, given our program’s implementation during the COVID-19 pandemic, we sought to assess the impact of the pandemic on the program participants. The results provide evidence that peers can use the information, motivation, and behavioral skills to facilitate engagement in HIV and STI testing and PrEP uptake. In addition, the provision of HIV and STI testing kits through a peer-based model can lead to increased STI testing and may increase PrEP use. Our results align with prior research that has demonstrated the effectiveness of peer-based interventions in shaping HIV-related behaviors in Latinx immigrant SMM [22-26]. Specifically, several studies have shown that peer education is effective in facilitating behavior change among groups at high risk of HIV globally [11].

The implementation of our peer-based intervention during the global pandemic was novel. As indicated by our results, Latinx immigrant SMM were greatly affected by the COVID-19 pandemic, with a significant number reporting job loss and distress. The social and economic effects of COVID-19 continue to be documented, demonstrating disproportionate impacts on immigrant communities in the United States [27]. Furthermore, HIV testing and screening for STIs were disrupted during the COVID-19 pandemic, with data pointing to substantial declines in HIV and STI testing rates from 2019 to 2020 [28-30].

As most participants (20/30, 67%) who received the STI and HIV self-testing kits completed and returned them with support from their project peers, our study demonstrates the potential benefits of using self-test and specimen self-collection technologies to enhance testing uptake. Specifically, our results showed that a greater proportion of the intervention group participants tested for STIs relative to the control group who did not receive the testing kits. Hence, although support and information from peers were useful in increasing motivation to get tested for HIV and STIs and use PrEP for both groups, receiving free self-testing kits may increase the likelihood of STI testing among Latinx immigrant SMM. These results are especially relevant in circumstances such as the COVID-19 pandemic, during which clinic closures and travel restrictions posed challenges. For our participants, self-collection STI testing using a free kit delivered directly to their homes might have been a vital means of overcoming access barriers. Although these results support our intervention, the costs of the kits and the feasibility of kit delivery and their use outside of a research study should be considered.

Reports of HIV testing after participating in the *Listos* program did not differ significantly between the study arms, which may have resulted from limited power in the sample. This finding may also be the result of public health efforts to ensure access to HIV testing. In contrast, relatively few interventions have sought to increase STI screening, and home testing interventions, in particular, have not previously been widely disseminated in this population. Our intervention may have provided many participants with the opportunity to learn about home-based STI testing for the first time. As national rates of STIs continue to increase and Hispanic and Latinx SMM experience a disproportionate burden of STIs [31], our study points to the urgent need to expand effective STI prevention and care programs that address gaps in service access. Our identification of one new HIV diagnosis and 3 new HSV-2 diagnoses highlights the opportunity for intervention to increase new HIV and STI diagnoses in the Latinx immigrant SMM community.

The intervention group was positively associated with motivation across the 3 behaviors (HIV testing, STI testing, and PrEP use) assessed, which suggests that the intervention group may have experienced more motivation compared with the control group. Having access to self-testing kits may have increased the accessibility of engaging in HIV testing behaviors, increasing motivation in the intervention group. This finding highlights that having direct access to HIV and STI testing services delivered by peers may be important for facilitating HIV prevention in Latinx immigrant SMM. By removing the need to visit a clinic to get tested for HIV and STIs, Latinx immigrant SMM may perceive testing behaviors and PrEP use as more acceptable.

Overall, most participants in both study arms reported that the *Listos* program motivated them to test for HIV and STIs and to use PrEP. Hence, our study underscores that peers are an important resource and can provide relevant information and motivate Latinx immigrant SMM to engage in HIV prevention behaviors. Although most participants (48/50, 96%) had previously been tested for HIV, a significant percentage had not used PrEP. Furthermore, more than half (28/50, 56%) of the participants did not have health insurance. As health insurance and language are major barriers to accessing health and HIV prevention services, particularly for immigrant and racial or ethnic minority communities, our study indicates that peers were able to overcome these challenges and reach Latinx immigrant SMM in ways that were acceptable and culturally sensitive during a major health crisis. However, costs and health insurance barriers remain important considerations for ensuring access to treatment and participation in sustained STI testing programs.

Peers were not only trained remotely but were also able to remotely deliver the *Listos* program to limit in-person contact during COVID-19. Although in-person intervention delivery may be more desirable for peer-based interventions, our study suggests that the integration of mobile technologies and web-based communication methods is invaluable for Latinx immigrant SMM in obtaining support, information, and resources. Furthermore, the use of web-based technology to train peers may increase opportunities to expand the capacity for delivering linguistically and culturally appropriate HIV prevention interventions for Latinx and immigrant communities.

### Limitations

This study has several limitations. First, participants were recruited by peers through their social networks and through web-based strategies using convenience sampling. Hence, the results cannot be generalized to all Latinx immigrant SMM. Peers may have known some of the participants previously, which may have biased the results. Second, because of challenges related to the COVID-19 pandemic, there was greater loss to follow-up than anticipated. Third, the small sample size limited our statistical analyses, which consequently could not be controlled for factors that differed across study arms. Therefore, our analyses require further confirmation. Fourth, because the peer-counseling component was delivered to both arms, we could not determine whether this component of the intervention had any impact on outcomes. In addition, we cannot attribute the uptake of self-testing kits to peers, given that both groups were exposed to peer counseling. Finally, the intervention was delivered in 2021, and the effects of the COVID-19 pandemic on access to health services should be considered when interpreting the results.

### Conclusions

By facilitating access to HIV and STI testing through HIV and STI self-testing kits and enhancing testing and PrEP information, motivation, behavioral skills, and empowerment through peers, our peer-based program demonstrated the potential to increase motivation to testing for HIV and STIs and using PrEP among Latinx immigrant SMM. Peer-based programs that offer self-testing kits and internet-based modes of accessing information may be a feasible and acceptable strategy to reach Latinx immigrant SMM and enhance HIV prevention and care behaviors when in-person services are limited or unavailable owing to conditions such as the COVID-19 pandemic. Future studies with long-term evaluations are needed to assess the costs and sustainability of such programs. In addition, future research may explore opportunities to integrate web-based technology to support and develop peer-based interventions to enhance HIV prevention and care for Latinx and immigrant communities.

## Appendix

Multimedia Appendix 1 CONSORT-eHEALTH checklist (V 1.6.1).

## Abbreviations

ART: antiretroviral therapy
HSV-2: herpes simplex virus type 2
PI: principal investigator
PrEP: preexposure prophylaxis
REDCap: Research Electronic Data Capture
SMM: sexual minority men
STI: sexually transmitted infection

## Data Availability

The data that support the findings of this study are available from the corresponding author on reasonable request. The data generated during this study are not publicly available because of the nature of this research and because the participants did not agree for their data to be shared publicly.

## References

[1] (2021). HIV surveillance report, 2019. Centers for Disease Control and Prevention.

[2] (2022). First year geographic focus, ending the HIV epidemic: a plan for America. Centers for Disease Control and Prevention.

[3] (2022). HIV/AIDS epidemiology report and community profile. Seattle & King County and the Infectious Disease Assessment Unit, Washington State Department of Health.

[4] (2022). PrEP (pre-exposure prophylaxis). Centers for Disease Control and Prevention.

[5] Guilamo-Ramos V, Thimm-Kaiser M, Benzekri A, Chacón G, López OR, Scaccabarrozzi L, Rios E (2020). The invisible US Hispanic/Latino HIV crisis: addressing gaps in the national response. Am J Public Health.

[6] (2021). HIV and Hispanic/Latino gay and bisexual men. Centers for Disease Control and Prevention.

[7] Shangani S, Escudero D, Kirwa K, Harrison A, Marshall B, Operario D (2017). Effectiveness of peer-led interventions to increase HIV testing among men who have sex with men: a systematic review and meta-analysis. AIDS Care.

[8] Simoni JM, Nelson KM, Franks JC, Yard SS, Lehavot K (2011). Are peer interventions for HIV efficacious? A systematic review. AIDS Behav.

[9] Medley A, Kennedy C, O'Reilly K, Sweat M (2009). Effectiveness of peer education interventions for HIV prevention in developing countries: a systematic review and meta-analysis. AIDS Educ Prev.

[10] Simoni JM, Franks JC, Lehavot K, Yard SS (2011). Peer interventions to promote health: conceptual considerations. Am J Orthopsychiatry.

[11] He J, Wang Y, Du Z, Liao J, He N, Hao Y (2020). Peer education for HIV prevention among high-risk groups: a systematic review and meta-analysis. BMC Infect Dis.

[12] Kersh EN, Shukla M, Raphael BH, Habel M, Park I (2021). At-home specimen self-collection and self-testing for sexually transmitted infection screening demand accelerated by the COVID-19 pandemic: a review of laboratory implementation issues. J Clin Microbiol.

[13] Stevens DR, Vrana CJ, Dlin RE, Korte JE (2018). A global review of HIV self-testing: themes and implications. AIDS Behav.

[14] Fisher WA, Fisher JD, Harman J, Suls J, Wallston KA (2003). The information-motivation-behavioral skills model: a general social psychological approach to understanding and promoting health behavior. Social Psychological Foundations of Health and Illness.

[15] Chang SJ, Choi S, Kim SA, Song M (2014). Intervention strategies based on information-motivation-behavioral skills model for health behavior change: a systematic review. Asian Nurs Res.

[16] Wang H, Chang R, Shen Q, Tsamlag L, Zhang S, Shi Y, Ma T, Wang Z, She R, Lau JT, Wang Y, Cai Y (2020). Information-motivation-behavioral skills model of consistent condom use among transgender women in Shenyang, China. BMC Public Health.

[17] Vindrola-Padros C, Johnson GA (2020). Rapid techniques in qualitative research: a critical review of the literature. Qual Health Res.

[18] St. George SM, Harkness AR, Rodriguez-Diaz CE, Weinstein ER, Pavia V, Hamilton AB (2023). Applying rapid qualitative analysis for health equity: lessons learned using “EARS” with Latino communities. Int J Qual Methods.

[19] Watkins DC (2017). Rapid and rigorous qualitative data analysis: the “RADaR” technique for applied research. Int J Qual Methods.

[20] (2022). COVID-19 timeline. Centers for Disease Control and Prevention.

[21] Harris PA, Taylor R, Thielke R, Payne J, Gonzalez N, Conde JG (2009). Research electronic data capture (REDCap)--a metadata-driven methodology and workflow process for providing translational research informatics support. J Biomed Inform.

[22] Rhodes SD, Hergenrather KC, Bloom FR, Leichliter JS, Montaño J (2009). Outcomes from a community-based, participatory lay health adviser HIV/STD prevention intervention for recently arrived immigrant Latino men in rural North Carolina. AIDS Educ Prev.

[23] Rhodes SD, Leichliter JS, Sun CJ, Bloom FR (2016). The HoMBReS and HoMBReS Por un Cambio interventions to reduce HIV disparities among immigrant Hispanic/Latino Men. MMWR Suppl.

[24] Johnson AK, Buenrostro R, Soberanis G, McCarn B, Magner B, Maiorana A (2021). Salud y Orgullo Mexicano: development of a culturally specific transnational linkage and retention in care intervention for Mexican men who have sex with men living with HIV in Chicago. J Immigr Minor Health (Forthcoming).

[25] Erausquin JT, Duan N, Grusky O, Swanson AN, Kerrone D, Rudy ET (2009). Increasing the reach of HIV testing to young Latino MSM: results of a pilot study integrating outreach and services. J Health Care Poor Underserved.

[26] Rhodes SD, Foley KL, Zometa CS, Bloom FR (2007). Lay health advisor interventions among Hispanics/Latinos: a qualitative systematic review. Am J Prev Med.

[27] Clark E, Fredricks K, Woc-Colburn L, Bottazzi ME, Weatherhead J (2020). Disproportionate impact of the COVID-19 pandemic on immigrant communities in the United States. PLoS Negl Trop Dis.

[28] Moitra E, Tao J, Olsen J, Shearer RD, Wood BR, Busch AM, LaPlante A, Baker JV, Chan PA (2022). Impact of the COVID-19 pandemic on HIV testing rates across four geographically diverse urban centres in the United States: an observational study. Lancet Reg Health Am.

[29] Pinto CN, Niles JK, Kaufman HW, Marlowe EM, Alagia DP, Chi G, Van Der Pol B (2021). Impact of the COVID-19 pandemic on chlamydia and gonorrhea screening in the U.S. Am J Prev Med.

[30] DiNenno EA, Delaney KP, Pitasi MA, MacGowan R, Miles G, Dailey A, Courtenay-Quirk C, Byrd K, Thomas D, Brooks JT, Daskalakis D, Collins N (2022). HIV testing before and during the COVID-19 pandemic - United States, 2019-2020. MMWR Morb Mortal Wkly Rep.

[31] (2021). Reported STDs reach all-time high for 6th consecutive year. Centers for Disease Control and Prevention.

