# Supplementary material for: A Peer-Based Intervention to Increase HIV and Sexually Transmitted Infection Testing Among Latinx Immigrant Sexual Minority Men in the US Pacific Northwest: Pilot Randomized Controlled Trial Conducted During the COVID-19 Pandemic
Source: JMIR Form Res. 2023 Jul 12;7:e45871. doi: 10.2196/45871 (PMC10372765; doi:10.2196/45871)
Supplement: Multimedia Appendix 1 [file formative_v7i1e45871_app1.pdf]

CONSORT-EHEALTH (V 1.6.1) - Submission/Publication Form

The CONSORT-EHEALTH checklist is intended for authors of randomized trials evaluating web-based and Internet-based applications/interventions, including mobile interventions, electronic games (incl multiplayer games), social media, certain telehealth applications, and other interactive and/or networked electronic applications. Some of the items (e.g. all subitems under item 5 - description of the intervention) may also be applicable for other study designs.

The goal of the CONSORT EHEALTH checklist and guideline is to be
a) a guide for reporting for authors of RCTs,
b) to form a basis for appraisal of an ehealth trial (in terms of validity)

CONSORT-EHEALTH items/subitems are MANDATORY reporting items for studies published in the Journal of Medical Internet Research and other journals / scientific societies endorsing the checklist.

Items numbered 1., 2., 3., 4a., 4b etc are original CONSORT or CONSORT-NPT (non-pharmacologic treatment) items.
Items with Roman numerals (i., ii, iii, iv etc.) are CONSORT-EHEALTH extensions/clarifications.

As the CONSORT-EHEALTH checklist is still considered in a formative stage, we would ask that you also RATE ON A SCALE OF 1-5 how important/useful you feel each item is FOR THE PURPOSE OF THE CHECKLIST and reporting guideline (optional).

Mandatory reporting items are marked with a red *.
In the textboxes, either copy & paste the relevant sections from your manuscript into this form - please include any quotes from your manuscript in QUOTATION MARKS,
or answer directly by providing additional information not in the manuscript, or elaborating on why the item was not relevant for this study.

YOUR ANSWERS WILL BE PUBLISHED AS A SUPPLEMENTARY FILE TO YOUR PUBLICATION IN JMIR AND ARE CONSIDERED PART OF YOUR PUBLICATION (IF ACCEPTED).
Please fill in these questions diligently. Information will not be copyedited, so please use proper spelling and grammar, use correct capitalization, and avoid abbreviations.

DO NOT FORGET TO SAVE AS PDF _AND_ CLICK THE SUBMIT BUTTON SO YOUR ANSWERS ARE IN OUR DATABASE !!!

Your response is too large. Try shortening some answers.

Eysenbach G, CONSORT-EHEALTH Group

CONSORT-EHEALTH: Improving and Standardizing Evaluation Reports of Web-based and Mobile Health Interventions
J Med Internet Res 2011;13(4):e126
URL: http://www.jmir.org/2011/4/e126/
doi: 10.2196/jmir.1923
PMID: 22209829

janejlee1205@gmail.com Switch account

Not shared

* Indicates required question

Your name *

First Last

Jane Lee

Primary Affiliation (short), City, Country *

University of Toronto, Toronto, Canada

University of Washington, Seattle, United State

Your e-mail address *

abc@gmail.com

janejlee@uw.edu

Your response is too large. Try shortening some answers.

Title of your manuscript *

Provide the (draft) title of your manuscript.

A Peer-Based Intervention to Increase HIV and Sexually Transmitted Infection Testing Among Latinx Immigrant Sexual Minority Men in the US Pacific Northwest: Pilot Randomized Controlled Trial Conducted During the COVID-19 Pandemic

Name of your App/Software/Intervention *

If there is a short and a long/alternate name, write the short name first and add the long name in brackets.

Listos [A Peer-based HIV and Sexually Transm

Evaluated Version (if any)

e.g. "V1", "Release 2017-03-01", "Version 2.0.27913"

Not Applicable

Language(s) *

What language is the intervention/app in? If multiple languages are available, separate by comma (e.g. "English, French")

Spanish, English

URL of your Intervention Website or App

e.g. a direct link to the mobile app on app in appstore (itunes, Google Play), or URL of the website. If the intervention is a DVD or hardware, you can also link to an Amazon page.

Your answer

Your response is too large. Try shortening some answers.

URL of an image/screenshot (optional)

Your answer

Accessibility *

Can an enduser access the intervention presently?

- ○ access is free and open
- ○ access only for special usergroups, not open
- ○ access is open to everyone, but requires payment/subscription/in-app purchases
- ◉ app/intervention no longer accessible
- ○ Other:

Primary Medical Indication/Disease/Condition *

e.g. "Stress", "Diabetes", or define the target group in brackets after the condition, e.g. "Autism (Parents of children with)", "Alzheimers (Informal Caregivers of)"

HIV (Latinx Sexual Minority Men)

Primary Outcomes measured in trial *

comma-separated list of primary outcomes reported in the trial

Having tested for HIV or STIs, PrEP use

Your response is too large. Try shortening some answers.

Secondary/other outcomes

Are there any other outcomes the intervention is expected to affect?

Motivation to test for HIV or STIs, Motivation to use PrEP

Recommended "Dose" *

What do the instructions for users say on how often the app should be used?

- ○ Approximately Daily
- ○ Approximately Weekly
- ○ Approximately Monthly
- ○ Approximately Yearly
- ○ "as needed"
- ◉ Other: The intervention did not included an app

Your response is too large. Try shortening some answers.

Approx. Percentage of Users (starters) still using the app as recommended after 3 months *

- (selected) unknown / not evaluated
- 0-10%
- 11-20%
- 21-30%
- 31-40%
- 41-50%
- 51-60%
- 61-70%
- 71%-80%
- 81-90%
- 91-100%
- Other:

Overall, was the app/intervention effective? *

- yes: all primary outcomes were significantly better in intervention group vs control
- (selected) partly: SOME primary outcomes were significantly better in intervention group vs control
- no statistically significant difference between control and intervention
- potentially harmful: control was significantly better than intervention in one or more outcomes
- inconclusive: more research is needed

Your response is too large. Try shortening some answers.

Article Preparation Status/Stage *

At which stage in your article preparation are you currently (at the time you fill in this form)

- ( ) not submitted yet - in early draft status
- ( ) not submitted yet - in late draft status, just before submission
- ( ) submitted to a journal but not reviewed yet
- ( ) submitted to a journal and after receiving initial reviewer comments
- (x) submitted to a journal and accepted, but not published yet
- ( ) published
- ( ) Other:

Journal *

If you already know where you will submit this paper (or if it is already submitted), please provide the journal name (if it is not JMIR, provide the journal name under "other")

- ( ) not submitted yet / unclear where I will submit this
- ( ) Journal of Medical Internet Research (JMIR)
- ( ) JMIR mHealth and UHealth
- ( ) JMIR Serious Games
- ( ) JMIR Mental Health
- ( ) JMIR Public Health
- (x) JMIR Formative Research
- ( ) Other JMIR sister journal
- ( ) Other:

Your response is too large. Try shortening some answers.

Is this a full powered effectiveness trial or a pilot/feasibility trial? *

- (●) Pilot/feasibility
- ( ) Fully powered

Manuscript tracking number *

If this is a JMIR submission, please provide the manuscript tracking number under "other" (The ms tracking number can be found in the submission acknowledgement email, or when you login as author in JMIR. If the paper is already published in JMIR, then the ms tracking number is the four-digit number at the end of the DOI, to be found at the bottom of each published article in JMIR)

- ( ) no ms number (yet) / not (yet) submitted to / published in JMIR
- (●) Other: 45871

TITLE AND ABSTRACT

1a) TITLE: Identification as a randomized trial in the title

1a) Does your paper address CONSORT item 1a? *

I.e does the title contain the phrase "Randomized Controlled Trial"? (if not, explain the reason under "other")

- (●) yes
- ( ) Other:

Your response is too large. Try shortening some answers.

1a-i) Identify the mode of delivery in the title

Identify the mode of delivery. Preferably use “web-based” and/or “mobile” and/or “electronic game” in the title. Avoid ambiguous terms like “online”, “virtual”, “interactive”. Use “Internet-based” only if Intervention includes non-web-based Internet components (e.g. email), use “computer-based” or “electronic” only if offline products are used. Use “virtual” only in the context of “virtual reality” (3-D worlds). Use “online” only in the context of “online support groups”. Complement or substitute product names with broader terms for the class of products (such as “mobile” or “smart phone” instead of “iphone”), especially if the application runs on different platforms.

subitem not at all important

1 ○

2 ○

3 ○

4 ○

5 ○

essential

Does your paper address subitem 1a-i? *

Copy and paste relevant sections from manuscript title (include quotes in quotation marks "like this" to indicate direct quotes from your manuscript), or elaborate on this item by providing additional information not in the ms, or briefly explain why the item is not applicable/relevant for your study

"Given the adaptation for the COVID-19 pandemic, peers communicated with participants via phone, email, text, and other internet-based communication methods (eg, Facetime and WhatsApp)."

Your response is too large. Try shortening some answers.

1a-ii) Non-web-based components or important co-interventions in title

Mention non-web-based components or important co-interventions in title, if any (e.g., “with telephone support”).

subitem not at all important

1 ○

2 ○

3 ○

4 ○

5 ○

essential

Does your paper address subitem 1a-ii?

Copy and paste relevant sections from manuscript title (include quotes in quotation marks "like this" to indicate direct quotes from your manuscript), or elaborate on this item by providing additional information not in the ms, or briefly explain why the item is not applicable/relevant for your study

"Peer-based"- the intervention was considered “peer-based” given that it was delivered by peers.

Your response is too large. Try shortening some answers.

1a-iii) Primary condition or target group in the title

Mention primary condition or target group in the title, if any (e.g., “for children with Type I Diabetes”) Example: A Web-based and Mobile Intervention with Telephone Support for Children with Type I Diabetes: Randomized Controlled Trial

subitem not at all important

1 ○

2 ○

3 ○

4 ○

5 ○

essential

Does your paper address subitem 1a-iii? *

Copy and paste relevant sections from manuscript title (include quotes in quotation marks "like this" to indicate direct quotes from your manuscript), or elaborate on this item by providing additional information not in the ms, or briefly explain why the item is not applicable/relevant for your study

"HIV and Sexually Transmitted Infection Testing Among Latinx Immigrant Sexual Minority Men"

1b) ABSTRACT: Structured summary of trial design, methods, results, and conclusions

NPT extension: Description of experimental treatment, comparator, care providers, centers, and blinding status.

Your response is too large. Try shortening some answers.

1b-i) Key features/functionalities/components of the intervention and comparator in the METHODS section of the ABSTRACT

Mention key features/functionalities/components of the intervention and comparator in the abstract. If possible, also mention theories and principles used for designing the site. Keep in mind the needs of systematic reviewers and indexers by including important synonyms. (Note: Only report in the abstract what the main paper is reporting. If this information is missing from the main body of text, consider adding it)

subitem not at all important

1 ○

2 ○

3 ○

4 ○

5 ○

essential

Does your paper address subitem 1b-i? *

Copy and paste relevant sections from the manuscript abstract (include quotes in quotation marks "like this" to indicate direct quotes from your manuscript), or elaborate on this item by providing additional information not in the ms, or briefly explain why the item is not applicable/relevant for your study

"We piloted the intervention with Latinx SMM and randomly assigned participants to the intervention group, who received peer counseling and HIV and STI self-testing kits, or to the control group, who only received peer counseling."

Your response is too large. Try shortening some answers.

1b-ii) Level of human involvement in the METHODS section of the ABSTRACT

Clarify the level of human involvement in the abstract, e.g., use phrases like “fully automated” vs. “therapist/nurse/care provider/physician-assisted” (mention number and expertise of providers involved, if any). (Note: Only report in the abstract what the main paper is reporting. If this information is missing from the main body of text, consider adding it)

subitem not at all important

1 ○

2 ○

3 ○

4 ○

5 ○

essential

Does your paper address subitem 1b-ii?

Copy and paste relevant sections from the manuscript abstract (include quotes in quotation marks "like this" to indicate direct quotes from your manuscript), or elaborate on this item by providing additional information not in the ms, or briefly explain why the item is not applicable/relevant for your study

"Owing to the COVID-19 pandemic, the intervention components were delivered via web-based modalities."

Your response is too large. Try shortening some answers.

1b-iii) Open vs. closed, web-based (self-assessment) vs. face-to-face assessments in the METHODS section of the ABSTRACT

Mention how participants were recruited (online vs. offline), e.g., from an open access website or from a clinic or a closed online user group (closed usergroup trial), and clarify if this was a purely web-based trial, or there were face-to-face components (as part of the intervention or for assessment). Clearly say if outcomes were self-assessed through questionnaires (as common in web-based trials). Note: In traditional offline trials, an open trial (open-label trial) is a type of clinical trial in which both the researchers and participants know which treatment is being administered. To avoid confusion, use "blinded" or "unblinded" to indicated the level of blinding instead of "open", as "open" in web-based trials usually refers to "open access" (i.e. participants can self-enrol). (Note: Only report in the abstract what the main paper is reporting. If this information is missing from the main body of text, consider adding it)

subitem not at all important

1 ○

2 ○

3 ○

4 ○

5 ○

essential

Does your paper address subitem 1b-iii?

Copy and paste relevant sections from the manuscript abstract (include quotes in quotation marks "like this" to indicate direct quotes from your manuscript), or elaborate on this item by providing additional information not in the ms, or briefly explain why the item is not applicable/relevant for your study

"We administered baseline, 1-week, 6-week, and 12-week follow-up surveys to assess behaviors related to HIV testing, STI testing, and PrEP uptake."

Your response is too large. Try shortening some answers.

1b-iv) RESULTS section in abstract must contain use data

Report number of participants enrolled/assessed in each group, the use/uptake of the intervention (e.g., attrition/adherence metrics, use over time, number of logins etc.), in addition to primary/secondary outcomes. (Note: Only report in the abstract what the main paper is reporting. If this information is missing from the main body of text, consider adding it)

subitem not at all important

1 ○

2 ○

3 ○

4 ○

5 ○

essential

Does your paper address subitem 1b-iv?

Copy and paste relevant sections from the manuscript abstract (include quotes in quotation marks "like this" to indicate direct quotes from your manuscript), or elaborate on this item by providing additional information not in the ms, or briefly explain why the item is not applicable/relevant for your study

"A total of 50 (30 in the intervention group and 20 in the control group) Latinx SMM participated in the program. Participants reported life disruptions owing to COVID-19, with 68% (34/50) reporting job loss after the declaration of the pandemic. After intervention participation, a higher proportion of participants in the intervention group reported having been tested for STIs (76% vs 36.8%; P=.01; Cramer V=0.394). Among the participants in the intervention group, 91% (21/23) reported being motivated to use PrEP compared with 59% (10/17) in the control group (P=.02; Cramer V=0.385)."

Your response is too large. Try shortening some answers.

1b-v) CONCLUSIONS/DISCUSSION in abstract for negative trials

Conclusions/Discussions in abstract for negative trials: Discuss the primary outcome - if the trial is negative (primary outcome not changed), and the intervention was not used, discuss whether negative results are attributable to lack of uptake and discuss reasons. (Note: Only report in the abstract what the main paper is reporting. If this information is missing from the main body of text, consider adding it)

subitem not at all important

1 ○

2 ○

3 ○

4 ○

5 ○

essential

Does your paper address subitem 1b-v?

Copy and paste relevant sections from the manuscript abstract (include quotes in quotation marks "like this" to indicate direct quotes from your manuscript), or elaborate on this item by providing additional information not in the ms, or briefly explain why the item is not applicable/relevant for your study

No, our manuscript abstract does not address this subitem because the primary outcome was not negative.

INTRODUCTION

2a) In INTRODUCTION: Scientific background and explanation of rationale

Your response is too large. Try shortening some answers.

2a-i) Problem and the type of system/solution

Describe the problem and the type of system/solution that is object of the study: intended as stand-alone intervention vs. incorporated in broader health care program? Intended for a particular patient population? Goals of the intervention, e.g., being more cost-effective to other interventions, replace or complement other solutions? (Note: Details about the intervention are provided in “Methods” under 5)

subitem not at all important

1 ○

2 ○

3 ○

4 ○

5 ○

essential

Does your paper address subitem 2a-i? *

Copy and paste relevant sections from the manuscript (include quotes in quotation marks "like this" to indicate direct quotes from your manuscript), or elaborate on this item by providing additional information not in the ms, or briefly explain why the item is not applicable/relevant for your study

"Given the potential of peer-based interventions and self-testing kits in addressing specific barriers to HIV prevention and care, we combined peer counseling and HIV and STI self-testing services to develop a peer-based HIV prevention intervention for Latinx immigrant SMM. The intervention was informed by the information-motivation-behavior skills model of health behavior change [14,15], which has extensive empirical support for the development of behavioral interventions in the context of HIV prevention with key populations at risk for HIV and among people with HIV taking ART [15,16]."

Your response is too large. Try shortening some answers.

2a-ii) Scientific background, rationale: What is known about the (type of) system

Scientific background, rationale: What is known about the (type of) system that is the object of the study (be sure to discuss the use of similar systems for other conditions/diagnoses, if appropiate), motivation for the study, i.e. what are the reasons for and what is the context for this specific study, from which stakeholder viewpoint is the study performed, potential impact of findings [2]. Briefly justify the choice of the comparator.

subitem not at all important

1 ◯

2 ◯

3 ◯

4 ◯

5 ◯

essential

Does your paper address subitem 2a-ii? *

Copy and paste relevant sections from the manuscript (include quotes in quotation marks "like this" to indicate direct quotes from your manuscript), or elaborate on this item by providing additional information not in the ms, or briefly explain why the item is not applicable/relevant for your study

"Peers provided counseling that included information about HIV and STI testing and PrEP, basic motivational interviewing for HIV prevention including PrEP among those testing negative, and the enhancement of behavioral skills to support engagement in HIV prevention services. The intervention also involved peer-supported delivery of HIV and STI self-testing kits for at-home specimen collection and submission, interpretation of results once available, and linkage to treatment, as indicated. We sought peers to address the cultural barriers, language issues, and stigma that pose challenges to HIV prevention and care among Latinx immigrant SMM. The use of self-testing kits was intended to facilitate the accessibility of HIV and STI testing for this population. Taken together, the combination of peer counseling and HIV and STI self-testing has the potential to overcome multiple structural-, social-, and individual-level barriers to HIV prevention and care."

Your response is too large. Try shortening some answers.

2b) In INTRODUCTION: Specific objectives or hypotheses

Does your paper address CONSORT subitem 2b? *

Copy and paste relevant sections from the manuscript (include quotes in quotation marks "like this" to indicate direct quotes from your manuscript), or elaborate on this item by providing additional information not in the ms, or briefly explain why the item is not applicable/relevant for your study

"The pilot trial examined the impact of the COVID-19 pandemic on study participants and differences in self-reports of HIV testing, STI testing, and PrEP uptake outcomes between the intervention and control group participants."

METHODS

3a) Description of trial design (such as parallel, factorial) including allocation ratio

Does your paper address CONSORT subitem 3a? *

Copy and paste relevant sections from the manuscript (include quotes in quotation marks "like this" to indicate direct quotes from your manuscript), or elaborate on this item by providing additional information not in the ms, or briefly explain why the item is not applicable/relevant for your study

"After the participants completed their baseline assessment, they were randomized to the intervention or control group using computer-generated simple randomization."
"After randomization, participants in both groups received their first individual counseling session with their peers, which lasted approximately 45 minutes. Participants in both arms also received follow-up sessions approximately 1 week before the 6- and 12-week surveys. Participants in both groups were able to access their peers via phone, email, or text throughout the course of the intervention. Only participants in the intervention group received HIV and STI testing kits, which were

Your response is too large. Try shortening some answers.

3b) Important changes to methods after trial commencement (such as eligibility criteria), with reasons

Does your paper address CONSORT subitem 3b? *

Copy and paste relevant sections from the manuscript (include quotes in quotation marks "like this" to indicate direct quotes from your manuscript), or elaborate on this item by providing additional information not in the ms, or briefly explain why the item is not applicable/relevant for your study

"Peers also used social media outreach strategies, such as messaging individuals in Facebook groups, to recruit potential participants. This recruitment strategy was a shift from initially planned efforts to recruit individuals using street intercept methods at community events, bars, and other social gathering locations."

3b-i) Bug fixes, Downtimes, Content Changes

Bug fixes, Downtimes, Content Changes: ehealth systems are often dynamic systems. A description of changes to methods therefore also includes important changes made on the intervention or comparator during the trial (e.g., major bug fixes or changes in the functionality or content) (5-iii) and other “unexpected events” that may have influenced study design such as staff changes, system failures/downtimes, etc. [2].

subitem not at all important

1 ○

2 ○

3 ○

4 ○

5 ○

essential

Your response is too large. Try shortening some answers.

Does your paper address subitem 3b-i?

Copy and paste relevant sections from the manuscript (include quotes in quotation marks "like this" to indicate direct quotes from your manuscript), or elaborate on this item by providing additional information not in the ms, or briefly explain why the item is not applicable/relevant for your study

"Before peer training in March 2020, the World Health Organization declared COVID-19 a pandemic, and US states began to shut down to prevent the spread of COVID-19 [20]. To protect the safety of peers and to follow local public health guidelines, all research activities were temporarily paused. We subsequently adapted the peer training and intervention procedures to be conducted virtually using internet and phone technologies. The 3-day peer training was conducted over Zoom (Zoom Video Communications), and all relevant materials and training supplies were mailed or

4a) Eligibility criteria for participants

Does your paper address CONSORT subitem 4a? *

Copy and paste relevant sections from the manuscript (include quotes in quotation marks "like this" to indicate direct quotes from your manuscript), or elaborate on this item by providing additional information not in the ms, or briefly explain why the item is not applicable/relevant for your study

"The eligibility criteria included (1) identify as Hispanic or Latinx, (2) immigrant or foreign born, (3) aged at least 18 years, (4) assigned male at birth, (5) report sex with men, and (6) and report unknown or HIV-negative status. We excluded participants who were known to be HIV positive and would therefore not be eligible for PrEP."

Your response is too large. Try shortening some answers.

4a-i) Computer / Internet literacy

Computer / Internet literacy is often an implicit “de facto” eligibility criterion - this should be explicitly clarified.

subitem not at all important

1 ○

2 ○

3 ○

4 ○

5 ○

essential

Does your paper address subitem 4a-i?

Copy and paste relevant sections from the manuscript (include quotes in quotation marks "like this" to indicate direct quotes from your manuscript), or elaborate on this item by providing additional information not in the ms, or briefly explain why the item is not applicable/relevant for your study

"Given the adaptation for the COVID-19 pandemic, peers communicated with participants via phone, email, text, and other internet-based communication methods (eg, Facetime and WhatsApp)."
"Specifically, peers walked participants through the completion of the self-testing kits via phone or video chat. Peers had sample kits to demonstrate and explain the testing procedures to the participants."

Your response is too large. Try shortening some answers.

4a-ii) Open vs. closed, web-based vs. face-to-face assessments:

Open vs. closed, web-based vs. face-to-face assessments: Mention how participants were recruited (online vs. offline), e.g., from an open access website or from a clinic, and clarify if this was a purely web-based trial, or there were face-to-face components (as part of the intervention or for assessment), i.e., to what degree got the study team to know the participant. In online-only trials, clarify if participants were quasi-anonymous and whether having multiple identities was possible or whether technical or logistical measures (e.g., cookies, email confirmation, phone calls) were used to detect/prevent these.

subitem not at all important

1 ○

2 ○

3 ○

4 ○

5 ○

essential

Does your paper address subitem 4a-ii? *

Copy and paste relevant sections from the manuscript (include quotes in quotation marks "like this" to indicate direct quotes from your manuscript), or elaborate on this item by providing additional information not in the ms, or briefly explain why the item is not applicable/relevant for your study

"Participants were recruited from February 2021 to December 2021 through peers' social networks, word-of-mouth, and referrals. Peers also used social media outreach strategies, such as messaging individuals in Facebook groups, to recruit potential participants. This recruitment strategy was a shift from initially planned efforts to recruit individuals using street intercept methods at community events, bars, and other

Your response is too large. Try shortening some answers.

4a-iii) Information giving during recruitment

Information given during recruitment. Specify how participants were briefed for recruitment and in the informed consent procedures (e.g., publish the informed consent documentation as appendix, see also item X26), as this information may have an effect on user self-selection, user expectation and may also bias results.

subitem not at all important

1 ○

2 ○

3 ○

4 ○

5 ○

essential

Does your paper address subitem 4a-iii?

Copy and paste relevant sections from the manuscript (include quotes in quotation marks "like this" to indicate direct quotes from your manuscript), or elaborate on this item by providing additional information not in the ms, or briefly explain why the item is not applicable/relevant for your study

"Assessments were conducted via web-based surveys at baseline (prerandomization) and at 1, 6, and 12 weeks post intervention. The surveys were self-administered using REDCap (Research Electronic Data Capture; Vanderbilt University) and were available in Spanish or English [21]. After the participants completed their baseline assessment, they were randomized to the intervention or control group using computer-generated simple randomization. Peers provided reminders about completing the surveys and offered support for survey completion. The survey assessments took approximately 30 to 60 minutes to complete."

4b) Settings and locations where the data were collected

Your response is too large. Try shortening some answers.

Does your paper address CONSORT subitem 4b? *

Copy and paste relevant sections from the manuscript (include quotes in quotation marks "like this" to indicate direct quotes from your manuscript), or elaborate on this item by providing additional information not in the ms, or briefly explain why the item is not applicable/relevant for your study

""Assessments were conducted via web-based surveys at baseline (prerandomization) and at 1, 6, and 12 weeks post intervention. The surveys were self-administered using REDCap (Research Electronic Data Capture; Vanderbilt University) and were available in Spanish or English [21]."

4b-i) Report if outcomes were (self-)assessed through online questionnaires

Clearly report if outcomes were (self-)assessed through online questionnaires (as common in web-based trials) or otherwise.

subitem not at all important

1 ○

2 ○

3 ○

4 ○

5 ○

essential

Your response is too large. Try shortening some answers.

Does your paper address subitem 4b-i? *

Copy and paste relevant sections from the manuscript (include quotes in quotation marks "like this" to indicate direct quotes from your manuscript), or elaborate on this item by providing additional information not in the ms, or briefly explain why the item is not applicable/relevant for your study

"Assessments were conducted via web-based surveys at baseline (prerandomization) and at 1, 6, and 12 weeks post intervention. The surveys were self-administered using REDCap (Research Electronic Data Capture; Vanderbilt University) and were available in Spanish or English [21]."

4b-ii) Report how institutional affiliations are displayed

Report how institutional affiliations are displayed to potential participants [on ehealth media], as affiliations with prestigious hospitals or universities may affect volunteer rates, use, and reactions with regards to an intervention.(Not a required item – describe only if this may bias results)

subitem not at all important

1 ○

2 ○

3 ○

4 ○

5 ○

essential

Does your paper address subitem 4b-ii?

Copy and paste relevant sections from the manuscript (include quotes in quotation marks "like this" to indicate direct quotes from your manuscript), or elaborate on this item by providing additional information not in the ms, or briefly explain why the item is not applicable/relevant for your study

Your response is too large. Try shortening some answers.

5) The interventions for each group with sufficient details to allow replication, including how and when they were actually administered

5-i) Mention names, credential, affiliations of the developers, sponsors, and owners

Mention names, credential, affiliations of the developers, sponsors, and owners [6] (if authors/evaluators are owners or developer of the software, this needs to be declared in a "Conflict of interest" section or mentioned elsewhere in the manuscript).

subitem not at all important

1 ○

2 ○

3 ○

4 ○

5 ○

essential

Your response is too large. Try shortening some answers.

Does your paper address subitem 5-i?

Copy and paste relevant sections from the manuscript (include quotes in quotation marks "like this" to indicate direct quotes from your manuscript), or elaborate on this item by providing additional information not in the ms, or briefly explain why the item is not applicable/relevant for your study

"Jane J Lee1, MSW, PhD; Gabriel Robles2, MSW, PhD; Christopher A Leyva Vera1, MSW, MPH; E Roberto Orellana1, MPhil, MPH, MSW, PhD; Susan M Graham3,4, MPH, PhD, MD; Anh-Minh Nguyen5, MS; Yingying Wei5, BS; Abraham Hernandez Sanchez1; Julia C Dombrowski3,6, MPH, MD; Jane Simoni4,7, MA, PhD"

"1School of Social Work, University of Washington, Seattle, WA, United States
2School of Social Work, Rutgers University, New Brunswick, NJ, United States
3Department of Medicine, University of Washington, Seattle, WA, United States
4Department of Global Health, University of Washington, Seattle, WA, United States
5Department of Biostatistics, University of Washington, Seattle, WA, United States
6HIV/STD Program, Public Health – Seattle & King County, Seattle, WA, United States
7Department of Psychology, University of Washington, Seattle, WA, United States"

5-ii) Describe the history/development process

Describe the history/development process of the application and previous formative evaluations (e.g., focus groups, usability testing), as these will have an impact on adoption/use rates and help with interpreting results.

subitem not at all important

1 ○

2 ○

3 ○

4 ○

5 ○

essential

Your response is too large. Try shortening some answers.

Does your paper address subitem 5-ii?

Copy and paste relevant sections from the manuscript (include quotes in quotation marks "like this" to indicate direct quotes from your manuscript), or elaborate on this item by providing additional information not in the ms, or briefly explain why the item is not applicable/relevant for your study

Your response is too large. Try shortening some answers.

"To elicit important factors to consider for training peers to deliver both education on HIV prevention and care and the HIV and STI self-testing intervention to Latinx immigrant SMM, we conducted semistructured in-depth interviews with community stakeholders (n=15) from diverse sectors, including community organizations (6/15, 40%), research (3/15, 20%), health care (4/15, 27%), and public health (2/15, 13%). Our objective in these interviews was to obtain perspectives from key stakeholders who are knowledgeable about HIV prevention in diverse Latinx populations to assess how best to work with peers and use HIV and STI self-testing as part of the intervention.
Interviews were facilitated by the study principal investigator (PI) and conducted over the phone or in-person based on the participant's availability and preference. As the purpose of the qualitative interviews was to inform processes for our intervention, we used rapid qualitative analysis to summarize key points and themes from the data [17,18]. Specifically, we used tables and spreadsheets to analyze the data and deductively identify themes that focused on specific barriers to HIV and STI testing and PrEP use in the Latinx immigrant SMM community and ways in which the planned intervention could help overcome these barriers [19]. The PI and 2 additional members of the research team discussed the final data tables for revision and agreement. The tables resulted in focused codes or themes that answered our overarching question of how to address barriers to HIV prevention through our intervention. Given that this qualitative analysis was central to the development of our intervention and informed our approach to piloting the intervention, we present the results of the qualitative interviews as part of the methods of our main study.
In-depth interviews revealed themes related to (1) HIV and STI testing knowledge, (2) PrEP knowledge, (3) access to HIV prevention and care services, (4) the acceptability and feasibility of HIV and STI self-testing, and (5) the role of peers in supporting the intervention. The interviews helped inform intervention development, as presented in Table 1. For example, stakeholders noted that cost and financial concerns are major barriers to accessing HIV prevention and care services, especially for men without access to medical insurance. Hence, we ensured that participation in the peer and HIV and STI testing intervention would be free of cost to the participants. In addition, stakeholders believed that familiarity with HIV testing and self-collection of STI specimens may be low among Latinx immigrant SMM. Thus, we integrated education on the skills needed to appropriately self-collect specimens for testing. Overall, the community stakeholder interviews validated the need for peer-based strategies for reaching Latinx immigrant SMM for HIV prevention. Several stakeholders explained how peers play critical roles in clinical and hospital settings to ensure that patients can make their appointments and navigate systems to access care and medication. They noted that this demonstrated the utility of peers in supporting Latinx SMM communities for facilitating HIV testing and PrEP uptake. Stakeholders also provided input on the types of HIV and STI tests to use and how to ensure appropriate follow-up and linkage

Your response is too large. Try shortening some answers.

5-iii) Revisions and updating

Revisions and updating. Clearly mention the date and/or version number of the application/intervention (and comparator, if applicable) evaluated, or describe whether the intervention underwent major changes during the evaluation process, or whether the development and/or content was “frozen” during the trial. Describe dynamic components such as news feeds or changing content which may have an impact on the replicability of the intervention (for unexpected events see item 3b).

subitem not at all important

1 ○

2 ○

3 ○

4 ○

5 ○

essential

Your response is too large. Try shortening some answers.

Does your paper address subitem 5-iii?

Copy and paste relevant sections from the manuscript (include quotes in quotation marks "like this" to indicate direct quotes from your manuscript), or elaborate on this item by providing additional information not in the ms, or briefly explain why the item is not applicable/relevant for your study

"The peer-based HIV and STI self-testing intervention was named Listos by community members during intervention development, which translates to “ready” or “smart.” The multiple meanings of the word were meant to highlight that Latinx SMM were not only ready for HIV prevention but were also smart for using HIV prevention services.
The intervention involved 2 main components. The first was peer counseling, which involved peers providing information about HIV and STI testing and PrEP, motivating participants to engage in HIV and STI testing and use PrEP, and reinforcing behavioral skills for HIV and STI testing and PrEP use. Peers also used empowerment strategies to help participants overcome the challenges in HIV prevention. The peer counseling session was conducted during the initial meeting between peers and participants after the baseline assessment. The intervention was considered “peer based,” given that it was delivered by peers. The second component was HIV and STI self-testing kits that were provided to the participants after the initial peer counseling session. On the basis of guidance from stakeholders, the intervention group received both intervention components, whereas the control group only received peer counseling.
For peer counseling, 3 separate modules were developed for peers to cover the participants during the initial individual session. The first involved providing relevant information about HIV and STI testing and PrEP, including what each entailed and their roles in preventing HIV transmission. The second involved a discussion about the reasons for testing for HIV and STI and using PrEP. Peers were to discuss the benefits of these HIV prevention behaviors and why an individual would get tested for HIV and STIs and use PrEP. Peers also discussed why HIV testing is key to linkage to ART initiation for undiagnosed people with HIV. The third module focused on relaying tailored information about how one would go about getting tested for HIV and STIs or using PrEP and reinforcing behavioral skills by providing step-by-step guidance on making appointments for HIV and STI testing and getting a prescription for PrEP."

Your response is too large. Try shortening some answers.

5-iv) Quality assurance methods

Provide information on quality assurance methods to ensure accuracy and quality of information provided [1], if applicable.

subitem not at all important

1 ○

2 ○

3 ○

4 ○

5 ○

essential

Does your paper address subitem 5-iv?

Copy and paste relevant sections from the manuscript (include quotes in quotation marks "like this" to indicate direct quotes from your manuscript), or elaborate on this item by providing additional information not in the ms, or briefly explain why the item is not applicable/relevant for your study

"Overall, peers were supported as experts in the community to be able to motivate and empower others to engage in HIV prevention behaviors. Peers checked in with participants before each follow-up assessment and provided additional counseling as needed, which was guided by the modules."

Your response is too large. Try shortening some answers.

5-v) Ensure replicability by publishing the source code, and/or providing screenshots/screen-capture video, and/or providing flowcharts of the algorithms used

Ensure replicability by publishing the source code, and/or providing screenshots/screen-capture video, and/or providing flowcharts of the algorithms used. Replicability (i.e., other researchers should in principle be able to replicate the study) is a hallmark of scientific reporting.

subitem not at all important

1 ○

2 ○

3 ○

4 ○

5 ○

essential

Does your paper address subitem 5-v?

Copy and paste relevant sections from the manuscript (include quotes in quotation marks "like this" to indicate direct quotes from your manuscript), or elaborate on this item by providing additional information not in the ms, or briefly explain why the item is not applicable/relevant for your study

N/A- Our paper does not have source code as there was no application or website used in the study.

Your response is too large. Try shortening some answers.

5-vi) Digital preservation

Digital preservation: Provide the URL of the application, but as the intervention is likely to change or disappear over the course of the years; also make sure the intervention is archived (Internet Archive, webcitation.org, and/or publishing the source code or screenshots/videos alongside the article). As pages behind login screens cannot be archived, consider creating demo pages which are accessible without login.

subitem not at all important

1 ○

2 ○

3 ○

4 ○

5 ○

essential

Does your paper address subitem 5-vi?

Copy and paste relevant sections from the manuscript (include quotes in quotation marks "like this" to indicate direct quotes from your manuscript), or elaborate on this item by providing additional information not in the ms, or briefly explain why the item is not applicable/relevant for your study

N/A- Our paper does not address this item as there was no application involved.

Your response is too large. Try shortening some answers.

5-vii) Access

Access: Describe how participants accessed the application, in what setting/context, if they had to pay (or were paid) or not, whether they had to be a member of specific group. If known, describe how participants obtained "access to the platform and Internet" [1]. To ensure access for editors/reviewers/readers, consider to provide a "backdoor" login account or demo mode for reviewers/readers to explore the application (also important for archiving purposes, see vi).

subitem not at all important

1 ○

2 ○

3 ○

4 ○

5 ○

essential

Your response is too large. Try shortening some answers.

Does your paper address subitem 5-vii? *

Copy and paste relevant sections from the manuscript (include quotes in quotation marks "like this" to indicate direct quotes from your manuscript), or elaborate on this item by providing additional information not in the ms, or briefly explain why the item is not applicable/relevant for your study

"The second component of the intervention involved HIV and STI self-testing kits that were provided to the participants after the initial peer counseling session. We used HIV and STI self-testing kits from Molecular Testing Labs that required self-collection of specimens and return mail submission for processing. The kits involved urine collection and blood spot collection using a lancet. Peers helped participants complete the self-collection kits by guiding them through the process and explaining how to use each of the items in the kit. Peers assisted the participants at each step and provided an overview of the instructions. The participants returned specimens via mail in postage-prepaid envelopes. Peers informed the participants of this step and reminded them to return the specimens. Molecular Testing Labs tested the specimens for HIV, Chlamydia trachomatis, Neisseria gonorrhea, Trichomonas vaginalis, hepatitis C antibody, herpes simplex virus type 2 (HSV-2), and syphilis. After returning the kits through mail, participants received their results via email within 7 days. Peers supported the participants in the interpretation of the test results once they were delivered to the participants. Peers connected participants who required additional follow-up care based on the test results to a local clinic, organization, or provider based

Your response is too large. Try shortening some answers.

5-viii) Mode of delivery, features/functionalities/components of the intervention and comparator, and the theoretical framework

Describe mode of delivery, features/functionalities/components of the intervention and comparator, and the theoretical framework [6] used to design them (instructional strategy [1], behaviour change techniques, persuasive features, etc., see e.g., [7, 8] for terminology). This includes an in-depth description of the content (including where it is coming from and who developed it) [1]," whether [and how] it is tailored to individual circumstances and allows users to track their progress and receive feedback" [6]. This also includes a description of communication delivery channels and – if computer-mediated communication is a component – whether communication was synchronous or asynchronous [6]. It also includes information on presentation strategies [1], including page design principles, average amount of text on pages, presence of hyperlinks to other resources, etc. [1].

subitem not at all important

1 ○

2 ○

3 ○

4 ○

5 ○

essential

Your response is too large. Try shortening some answers.

Does your paper address subitem 5-viii? *

Copy and paste relevant sections from the manuscript (include quotes in quotation marks "like this" to indicate direct quotes from your manuscript), or elaborate on this item by providing additional information not in the ms, or briefly explain why the item is not applicable/relevant for your study

Your response is too large. Try shortening some answers.

"The intervention involved 2 main components. The first was peer counseling, which involved peers providing information about HIV and STI testing and PrEP, motivating participants to engage in HIV and STI testing and use PrEP, and reinforcing behavioral skills for HIV and STI testing and PrEP use. Peers also used empowerment strategies to help participants overcome the challenges in HIV prevention. The peer counseling session was conducted during the initial meeting between peers and participants after the baseline assessment. The intervention was considered “peer based,” given that it was delivered by peers. The second component was HIV and STI self-testing kits that were provided to the participants after the initial peer counseling session. On the basis of guidance from stakeholders, the intervention group received both intervention components, whereas the control group only received peer counseling.
For peer counseling, 3 separate modules were developed for peers to cover the participants during the initial individual session. The first involved providing relevant information about HIV and STI testing and PrEP, including what each entailed and their roles in preventing HIV transmission. The second involved a discussion about the reasons for testing for HIV and STI and using PrEP. Peers were to discuss the benefits of these HIV prevention behaviors and why an individual would get tested for HIV and STIs and use PrEP. Peers also discussed why HIV testing is key to linkage to ART initiation for undiagnosed people with HIV. The third module focused on relaying tailored information about how one would go about getting tested for HIV and STIs or using PrEP and reinforcing behavioral skills by providing step-by-step guidance on making appointments for HIV and STI testing and getting a prescription for PrEP. Overall, peers were supported as experts in the community to be able to motivate and empower others to engage in HIV prevention behaviors. Peers checked in with participants before each follow-up assessment and provided additional counseling as needed, which was guided by the modules.
The second component of the intervention involved HIV and STI self-testing kits that were provided to the participants after the initial peer counseling session. We used HIV and STI self-testing kits from Molecular Testing Labs that required self-collection of specimens and return mail submission for processing. The kits involved urine collection and blood spot collection using a lancet. Peers helped participants complete the self-collection kits by guiding them through the process and explaining how to use each of the items in the kit. Peers assisted the participants at each step and provided an overview of the instructions. The participants returned specimens via mail in postage-prepaid envelopes. Peers informed the participants of this step and reminded them to return the specimens. Molecular Testing Labs tested the specimens for HIV, Chlamydia trachomatis, Neisseria gonorrhea, Trichomonas vaginalis, hepatitis C antibody, herpes simplex virus type 2 (HSV-2), and syphilis. After returning the kits through mail, participants received their results via email within 7 days. Peers supported the participants in the interpretation of the test results once they were delivered to the participants. Peers connected participants who required additional follow-up care based on the test results to a local clinic, organization, or provider based

Your response is too large. Try shortening some answers.

5-ix) Describe use parameters

Describe use parameters (e.g., intended “doses” and optimal timing for use). Clarify what instructions or recommendations were given to the user, e.g., regarding timing, frequency, heaviness of use, if any, or was the intervention used ad libitum.

subitem not at all important

1 ○

2 ○

3 ○

4 ○

5 ○

essential

Your response is too large. Try shortening some answers.

Does your paper address subitem 5-ix?

Copy and paste relevant sections from the manuscript (include quotes in quotation marks "like this" to indicate direct quotes from your manuscript), or elaborate on this item by providing additional information not in the ms, or briefly explain why the item is not applicable/relevant for your study

Your response is too large. Try shortening some answers.

"The intervention involved 2 main components. The first was peer counseling, which involved peers providing information about HIV and STI testing and PrEP, motivating participants to engage in HIV and STI testing and use PrEP, and reinforcing behavioral skills for HIV and STI testing and PrEP use. Peers also used empowerment strategies to help participants overcome the challenges in HIV prevention. The peer counseling session was conducted during the initial meeting between peers and participants after the baseline assessment. The intervention was considered “peer based,” given that it was delivered by peers. The second component was HIV and STI self-testing kits that were provided to the participants after the initial peer counseling session. On the basis of guidance from stakeholders, the intervention group received both intervention components, whereas the control group only received peer counseling.
For peer counseling, 3 separate modules were developed for peers to cover the participants during the initial individual session. The first involved providing relevant information about HIV and STI testing and PrEP, including what each entailed and their roles in preventing HIV transmission. The second involved a discussion about the reasons for testing for HIV and STI and using PrEP. Peers were to discuss the benefits of these HIV prevention behaviors and why an individual would get tested for HIV and STIs and use PrEP. Peers also discussed why HIV testing is key to linkage to ART initiation for undiagnosed people with HIV. The third module focused on relaying tailored information about how one would go about getting tested for HIV and STIs or using PrEP and reinforcing behavioral skills by providing step-by-step guidance on making appointments for HIV and STI testing and getting a prescription for PrEP. Overall, peers were supported as experts in the community to be able to motivate and empower others to engage in HIV prevention behaviors. Peers checked in with participants before each follow-up assessment and provided additional counseling as needed, which was guided by the modules.
The second component of the intervention involved HIV and STI self-testing kits that were provided to the participants after the initial peer counseling session. We used HIV and STI self-testing kits from Molecular Testing Labs that required self-collection of specimens and return mail submission for processing. The kits involved urine collection and blood spot collection using a lancet. Peers helped participants complete the self-collection kits by guiding them through the process and explaining how to use each of the items in the kit. Peers assisted the participants at each step and provided an overview of the instructions. The participants returned specimens via mail in postage-prepaid envelopes. Peers informed the participants of this step and reminded them to return the specimens. Molecular Testing Labs tested the specimens for HIV, Chlamydia trachomatis, Neisseria gonorrhea, Trichomonas vaginalis, hepatitis C antibody, herpes simplex virus type 2 (HSV-2), and syphilis. After returning the kits through mail, participants received their results via email within 7 days. Peers supported the participants in the interpretation of the test results once they were delivered to the participants. Peers connected participants who required additional follow-up care based on the test results to a local clinic, organization, or provider based

Your response is too large. Try shortening some answers.

5-x) Clarify the level of human involvement

Clarify the level of human involvement (care providers or health professionals, also technical assistance) in the e-intervention or as co-intervention (detail number and expertise of professionals involved, if any, as well as “type of assistance offered, the timing and frequency of the support, how it is initiated, and the medium by which the assistance is delivered”. It may be necessary to distinguish between the level of human involvement required for the trial, and the level of human involvement required for a routine application outside of a RCT setting (discuss under item 21 – generalizability).

subitem not at all important

1 ○

2 ○

3 ○

4 ○

5 ○

essential

Your response is too large. Try shortening some answers.

Does your paper address subitem 5-x?

Copy and paste relevant sections from the manuscript (include quotes in quotation marks "like this" to indicate direct quotes from your manuscript), or elaborate on this item by providing additional information not in the ms, or briefly explain why the item is not applicable/relevant for your study

"Given the adaptation for the COVID-19 pandemic, peers communicated with participants via phone, email, text, and other internet-based communication methods (eg, Facetime and WhatsApp). After randomization, participants in both groups received their first individual counseling session with their peers, which lasted approximately 45 minutes. Participants in both arms also received follow-up sessions approximately 1 week before the 6- and 12-week surveys. Participants in both groups were able to access their peers via phone, email, or text throughout the course of the intervention. Only participants in the intervention group received HIV and STI testing kits, which were mailed to their homes after randomization. For these participants, kits were registered on their behalf by the study PI, and peers provided internet-based support for completing the kits as part of the initial peer counseling session. Specifically, peers walked participants through the completion of the self-testing kits via phone or video chat. Peers had sample kits to demonstrate and explain the testing

5-xi) Report any prompts/reminders used

Report any prompts/reminders used: Clarify if there were prompts (letters, emails, phone calls, SMS) to use the application, what triggered them, frequency etc. It may be necessary to distinguish between the level of prompts/reminders required for the trial, and the level of prompts/reminders for a routine application outside of a RCT setting (discuss under item 21 – generalizability).

subitem not at all important

1 ○

2 ○

3 ○

4 ○

5 ○

essential

Your response is too large. Try shortening some answers.

Does your paper address subitem 5-xi? *

Copy and paste relevant sections from the manuscript (include quotes in quotation marks "like this" to indicate direct quotes from your manuscript), or elaborate on this item by providing additional information not in the ms, or briefly explain why the item is not applicable/relevant for your study

"Peers assisted the participants at each step and provided an overview of the instructions. The participants returned specimens via mail in postage-prepaid envelopes. Peers informed the participants of this step and reminded them to return the specimens. Molecular Testing Labs tested the specimens for HIV, Chlamydia trachomatis, Neisseria gonorrhea, Trichomonas vaginalis, hepatitis C antibody, herpes simplex virus type 2 (HSV-2), and syphilis. After returning the kits through mail, participants received their results via email within 7 days. Peers supported the participants in the interpretation of the test results once they were delivered to the participants. Peers connected participants who required additional follow-up care based on the test results to a local clinic, organization, or provider based on the

5-xii) Describe any co-interventions (incl. training/support)

Describe any co-interventions (incl. training/support): Clearly state any interventions that are provided in addition to the targeted eHealth intervention, as ehealth intervention may not be designed as stand-alone intervention. This includes training sessions and support [1]. It may be necessary to distinguish between the level of training required for the trial, and the level of training for a routine application outside of a RCT setting (discuss under item 21 – generalizability.

subitem not at all important

1 ○

2 ○

3 ○

4 ○

5 ○

essential

Your response is too large. Try shortening some answers.

Does your paper address subitem 5-xii? *

Copy and paste relevant sections from the manuscript (include quotes in quotation marks "like this" to indicate direct quotes from your manuscript), or elaborate on this item by providing additional information not in the ms, or briefly explain why the item is not applicable/relevant for your study

"Peers for the project met the following characteristics, which were also required of the study participants: (1) identify as Hispanic or Latinx, (2) immigrant or foreign born, (3) aged at least 18 years, (4) assigned male at birth, and (5) report sex with men. HIV status was not an exclusion criterion for participation as a peer. Peers were recruited through referrals from community-based organizations and linkages from prior research studies. Peers were interviewed and selected based on their interest in helping the community, experience with and understanding of HIV and STI prevention, and aptitude for supporting others to engage in HIV prevention behaviors. Peers were considered part of the study team and were compensated as consultants for their work in the study. Five peers were recruited to implement the intervention. The average age of the peers was 34 (SD 7.0; range 24-41) years, and the average duration of time residing in the United States was 10 (SD 5.5; range 2-16) years. A total of 4 peers were born in Mexico, and 1 of the peers was born in Honduras.

Before peer training in March 2020, the World Health Organization declared COVID-19 a pandemic, and US states began to shut down to prevent the spread of COVID-19 [20]. To protect the safety of peers and to follow local public health guidelines, all research activities were temporarily paused. We subsequently adapted the peer training and intervention procedures to be conducted virtually using internet and phone technologies. The 3-day peer training was conducted over Zoom (Zoom Video Communications), and all relevant materials and training supplies were mailed or emailed to peers.

The training sought to support peers in delivering the intervention with fidelity. At the end of the training, the facilitator conducted mock sessions with each of the peers to ensure that they were knowledgeable about all aspects of the intervention and study protocol, covered each of the content areas, provided correct and adequate information, and used appropriate techniques to motivate and empower participants. The PI maintained regular contact and follow-up with peers to support fidelity in the delivery of the intervention. Specifically, the PI supervised all peers, which included

6a) Completely defined pre-specified primary and secondary outcome measures, including how and when they were assessed

Your response is too large. Try shortening some answers.

Does your paper address CONSORT subitem 6a? *

Copy and paste relevant sections from the manuscript (include quotes in quotation marks "like this" to indicate direct quotes from your manuscript), or elaborate on this item by providing additional information not in the ms, or briefly explain why the item is not applicable/relevant for your study

"Baseline surveys asked participants about their HIV testing behaviors (ever tested for HIV and time since the last HIV test) and the result of their last HIV test if they had ever tested for HIV. Baseline surveys also asked about their STI testing behaviors (whether they had ever tested for gonorrhea, chlamydia, syphilis, hepatitis B, hepatitis C, genital warts, and genital herpes) and the result of their last STI test if they had ever been tested for any STI. PrEP use behaviors were also assessed at baseline with questions that asked whether participants were aware of PrEP, whether they had talked with their health care provider about PrEP, whether they had used PrEP in the last 12 months, and whether they were currently taking PrEP. Added questions at baseline and follow-ups owing to the COVID-19 pandemic included the experience of COVID-19 symptoms, COVID-19 testing and test results, and job loss. The impact of COVID-19 on participants was assessed by a question that asked how much COVID-19 affected their day-to-day lives on a scale of 1="not at all" to 5="an extreme amount." Distress related to COVID-19 was assessed by a question that asked, "In general, how much distress have you experienced in relation to COVID-19," with response options ranging on a scale of 1="no distress" to 10="extreme distress." Finally, participants indicated whether they were scared of being diagnosed with COVID-19 (yes, no, or do not know).

At each follow-up assessment, participants were asked about their HIV testing behaviors, STI testing behaviors, and PrEP use behaviors since the baseline assessment. Participants were also asked whether participating in the program motivated them to get tested for HIV or for STIs since the baseline assessment (yes or no). Finally, participants reported whether they had started taking PrEP since the completion of the baseline assessment and whether participation in the program motivated them to use PrEP.

For the intervention group participants who received HIV and STI self-testing kits, we received information from Molecular Testing Labs regarding whether they received completed testing kits. Participants who completed the testing kits received the results through email. The PI also sent these results to ensure that peers reviewed the results with participants and ensured that there was appropriate linkage to follow-up care. The location at which care was provided depended on the location of the participant and

Your response is too large. Try shortening some answers.

6a-i) Online questionnaires: describe if they were validated for online use and apply CHERRIES items to describe how the questionnaires were designed/deployed

If outcomes were obtained through online questionnaires, describe if they were validated for online use and apply CHERRIES items to describe how the questionnaires were designed/deployed [9].

subitem not at all important

1 ○

2 ○

3 ○

4 ○

5 ○

essential

Does your paper address subitem 6a-i?

Copy and paste relevant sections from manuscript text

Our paper does not address subitem 6a-i. In our manuscript, we note, "Assessments were conducted via web-based surveys at baseline (prerandomization) and at 1, 6, and 12 weeks post intervention. The surveys were self-administered using REDCap (Research Electronic Data Capture; Vanderbilt University) and were available in Spanish or English [21]. After the participants completed their baseline assessment, they were randomized to the intervention or control group using computer-generated simple randomization. Peers provided reminders about completing the surveys and offered support for survey completion. The survey assessments took approximately 30 to 60

Your response is too large. Try shortening some answers.

6a-ii) Describe whether and how "use" (including intensity of use/dosage) was defined/measured/monitored

Describe whether and how "use" (including intensity of use/dosage) was defined/measured/monitored (logins, logfile analysis, etc.). Use/adoption metrics are important process outcomes that should be reported in any ehealth trial.

subitem not at all important

1 ○

2 ○

3 ○

4 ○

5 ○

essential

Does your paper address subitem 6a-ii?

Copy and paste relevant sections from manuscript text

Our paper does not address this subitem given that there was no intensity of use or dosage involved.

Your response is too large. Try shortening some answers.

6a-iii) Describe whether, how, and when qualitative feedback from participants was obtained

Describe whether, how, and when qualitative feedback from participants was obtained (e.g., through emails, feedback forms, interviews, focus groups).

subitem not at all important

1 ○

2 ○

3 ○

4 ○

5 ○

essential

Does your paper address subitem 6a-iii?

Copy and paste relevant sections from manuscript text

"...peers communicated with participants via phone, email, text, and other internet-based communication methods (eg, Facetime and WhatsApp)."

6b) Any changes to trial outcomes after the trial commenced, with reasons

Does your paper address CONSORT subitem 6b? *

Copy and paste relevant sections from the manuscript (include quotes in quotation marks "like this" to indicate direct quotes from your manuscript), or elaborate on this item by providing additional information not in the ms, or briefly explain why the item is not applicable/relevant for your study

There were no changes to trial outcomes after the trial commenced.

Your response is too large. Try shortening some answers.

7a) How sample size was determined

NPT: When applicable, details of whether and how the clustering by care provides or centers was addressed

7a-i) Describe whether and how expected attrition was taken into account when calculating the sample size

Describe whether and how expected attrition was taken into account when calculating the sample size.

subitem not at all important

1 ○

2 ○

3 ○

4 ○

5 ○

essential

Does your paper address subitem 7a-i?

Copy and paste relevant sections from manuscript title (include quotes in quotation marks "like this" to indicate direct quotes from your manuscript), or elaborate on this item by providing additional information not in the ms, or briefly explain why the item is not applicable/relevant for your study

"As this was a pilot study, the analyses were not powered to detect the intervention effects. Study analyses to assess preliminary intervention efficacy were based on the intent-to-treat sample, and all randomized participants were included in the analyses."

7b) When applicable, explanation of any interim analyses and stopping guidelines

Your response is too large. Try shortening some answers.

Does your paper address CONSORT subitem 7b? *

Copy and paste relevant sections from the manuscript (include quotes in quotation marks "like this" to indicate direct quotes from your manuscript), or elaborate on this item by providing additional information not in the ms, or briefly explain why the item is not applicable/relevant for your study

We do not address this subitem. There were no interim analyses.

8a) Method used to generate the random allocation sequence
NPT: When applicable, how care providers were allocated to each trial group

Does your paper address CONSORT subitem 8a? *

Copy and paste relevant sections from the manuscript (include quotes in quotation marks "like this" to indicate direct quotes from your manuscript), or elaborate on this item by providing additional information not in the ms, or briefly explain why the item is not applicable/relevant for your study

"After the participants completed their baseline assessment, they were randomized to the intervention or control group using computer-generated simple randomization."

8b) Type of randomisation; details of any restriction (such as blocking and block size)

Does your paper address CONSORT subitem 8b? *

Copy and paste relevant sections from the manuscript (include quotes in quotation marks "like this" to indicate direct quotes from your manuscript), or elaborate on this item by providing additional information not in the ms, or briefly explain why the item is not applicable/relevant for your study

"After the participants completed their baseline assessment, they were randomized to the intervention or control group using computer-generated simple randomization."

Your response is too large. Try shortening some answers.

9) Mechanism used to implement the random allocation sequence (such as sequentially numbered containers), describing any steps taken to conceal the sequence until interventions were assigned

Does your paper address CONSORT subitem 9? *

Copy and paste relevant sections from the manuscript (include quotes in quotation marks "like this" to indicate direct quotes from your manuscript), or elaborate on this item by providing additional information not in the ms, or briefly explain why the item is not applicable/relevant for your study

"After the participants completed their baseline assessment, they were randomized to the intervention or control group using computer-generated simple randomization. Peers provided reminders about completing the surveys and offered support for survey completion. The survey assessments took approximately 30 to 60 minutes to complete. Given the adaptation for the COVID-19 pandemic, peers communicated with participants via phone, email, text, and other internet-based communication methods (eg, Facetime and WhatsApp). After randomization, participants in both groups received their first individual counseling session with their peers, which lasted approximately 45 minutes. Participants in both arms also received follow-up sessions approximately 1 week before the 6- and 12-week surveys. Participants in both groups were able to access their peers via phone, email, or text throughout the course of the

10) Who generated the random allocation sequence, who enrolled participants, and who assigned participants to interventions

Your response is too large. Try shortening some answers.

Does your paper address CONSORT subitem 10? *

Copy and paste relevant sections from the manuscript (include quotes in quotation marks "like this" to indicate direct quotes from your manuscript), or elaborate on this item by providing additional information not in the ms, or briefly explain why the item is not applicable/relevant for your study

"After the participants completed their baseline assessment, they were randomized to the intervention or control group using computer-generated simple randomization. Peers provided reminders about completing the surveys and offered support for survey completion."
"After the participants completed their baseline assessment, they were randomized to the intervention or control group using computer-generated simple randomization. Peers provided reminders about completing the surveys and offered support for survey completion. The survey assessments took approximately 30 to 60 minutes to complete. Given the adaptation for the COVID-19 pandemic, peers communicated with participants via phone, email, text, and other internet-based communication methods (eg, Facetime and WhatsApp). After randomization, participants in both groups received their first individual counseling session with their peers, which lasted approximately 45 minutes. Participants in both arms also received follow-up sessions approximately 1 week before the 6- and 12-week surveys. Participants in both groups were able to access their peers via phone, email, or text throughout the course of the

11a) If done, who was blinded after assignment to interventions (for example, participants, care providers, those assessing outcomes) and how

NPT: Whether or not administering co-interventions were blinded to group assignment

Your response is too large. Try shortening some answers.

11a-i) Specify who was blinded, and who wasn't

Specify who was blinded, and who wasn't. Usually, in web-based trials it is not possible to blind the participants [1, 3] (this should be clearly acknowledged), but it may be possible to blind outcome assessors, those doing data analysis or those administering co-interventions (if any).

subitem not at all important

1 ○

2 ○

3 ○

4 ○

5 ○

essential

Does your paper address subitem 11a-i? *

Copy and paste relevant sections from the manuscript (include quotes in quotation marks "like this" to indicate direct quotes from your manuscript), or elaborate on this item by providing additional information not in the ms, or briefly explain why the item is not applicable/relevant for your study

No participants were blinded.

Your response is too large. Try shortening some answers.

11a-ii) Discuss e.g., whether participants knew which intervention was the “intervention of interest” and which one was the “comparator”

Informed consent procedures (4a-ii) can create biases and certain expectations - discuss e.g., whether participants knew which intervention was the “intervention of interest” and which one was the “comparator”.

subitem not at all important

1 ○

2 ○

3 ○

4 ○

5 ○

essential

Does your paper address subitem 11a-ii?

Copy and paste relevant sections from the manuscript (include quotes in quotation marks "like this" to indicate direct quotes from your manuscript), or elaborate on this item by providing additional information not in the ms, or briefly explain why the item is not applicable/relevant for your study

Your answer

11b) If relevant, description of the similarity of interventions

(this item is usually not relevant for ehealth trials as it refers to similarity of a placebo or sham intervention to a active medication/intervention)

Your response is too large. Try shortening some answers.

Does your paper address CONSORT subitem 11b? *

Copy and paste relevant sections from the manuscript (include quotes in quotation marks "like this" to indicate direct quotes from your manuscript), or elaborate on this item by providing additional information not in the ms, or briefly explain why the item is not applicable/relevant for your study

"After randomization, participants in both groups received their first individual counseling session with their peers, which lasted approximately 45 minutes. Participants in both arms also received follow-up sessions approximately 1 week before the 6- and 12-week surveys. Participants in both groups were able to access their peers via phone, email, or text throughout the course of the intervention. Only participants in the intervention group received HIV and STI testing kits, which were mailed to their homes after randomization. For these participants, kits were registered on their behalf by the study PI, and peers provided internet-based support for completing the kits as part of the initial peer counseling session. Specifically, peers walked participants through the completion of the self-testing kits via phone or video chat. Peers had sample kits to demonstrate and explain the testing procedures to the

12a) Statistical methods used to compare groups for primary and secondary outcomes

NPT: When applicable, details of whether and how the clustering by care providers or centers was addressed

Your response is too large. Try shortening some answers.

Does your paper address CONSORT subitem 12a? *

Copy and paste relevant sections from the manuscript (include quotes in quotation marks "like this" to indicate direct quotes from your manuscript), or elaborate on this item by providing additional information not in the ms, or briefly explain why the item is not applicable/relevant for your study

"To assess the differences in self-reported outcomes related to HIV testing, STI testing, and PrEP uptake between the intervention and control groups after program participation, we performed 2-sided $\chi 2$ tests at .05 significance level. Cramer V was used to measure the strength of the association between the intervention condition (Listos intervention or control) and each outcome variable. Cramer V values range from 0 to 1, with values closer to 1 indicating a stronger association. As this was a pilot study, the analyses were not powered to detect the intervention effects. Study analyses to assess preliminary intervention efficacy were based on the intent-to-treat sample, and all randomized participants were included in the analyses. Given the small sample sizes and incomplete follow-up data for all participants, we combined responses to all follow-up assessments to determine differences by the 12-week assessment point. Specifically, a last observation carried forward approach was used, where missing follow-up data were replaced by the participant's previously observed value."

12a-i) Imputation techniques to deal with attrition / missing values

Imputation techniques to deal with attrition / missing values: Not all participants will use the intervention/comparator as intended and attrition is typically high in ehealth trials. Specify how participants who did not use the application or dropped out from the trial were treated in the statistical analysis (a complete case analysis is strongly discouraged, and simple imputation techniques such as LOCF may also be problematic [4]).

subitem not at all important

1 ○

2 ○

3 ○

4 ○

5 ○

essential

Your response is too large. Try shortening some answers.

Does your paper address subitem 12a-i? *

Copy and paste relevant sections from the manuscript (include quotes in quotation marks "like this" to indicate direct quotes from your manuscript), or elaborate on this item by providing additional information not in the ms, or briefly explain why the item is not applicable/relevant for your study

"As this was a pilot study, the analyses were not powered to detect the intervention effects. Study analyses to assess preliminary intervention efficacy were based on the intent-to-treat sample, and all randomized participants were included in the analyses. Given the small sample sizes and incomplete follow-up data for all participants, we combined responses to all follow-up assessments to determine differences by the 12-week assessment point. Specifically, a last observation carried forward approach was used, where missing follow-up data were replaced by the participant's previously observed value."

12b) Methods for additional analyses, such as subgroup analyses and adjusted analyses

Does your paper address CONSORT subitem 12b? *

Copy and paste relevant sections from the manuscript (include quotes in quotation marks "like this" to indicate direct quotes from your manuscript), or elaborate on this item by providing additional information not in the ms, or briefly explain why the item is not applicable/relevant for your study

"We calculated descriptive statistics for all variables, including those related to COVID-19 and tested for differences between the intervention and control groups at baseline using Fisher exact tests or t tests to ensure that the groups were appropriately

X26) REB/IRB Approval and Ethical Considerations [recommended as subheading under "Methods"] (not a CONSORT item)

Your response is too large. Try shortening some answers.

X26-i) Comment on ethics committee approval

subitem not at all important

1 ○

2 ○

3 ○

4 ○

5 ○

essential

Does your paper address subitem X26-i?

Copy and paste relevant sections from the manuscript (include quotes in quotation marks "like this" to indicate direct quotes from your manuscript), or elaborate on this item by providing additional information not in the ms, or briefly explain why the item is not applicable/relevant for your study

"All study procedures were reviewed and approved by the Institutional Review Board at the University of Washington under FWA #00006878 (STUDY00007054). All participants provided informed consent before participating in the study. The participants received a US $35 electronic gift card incentive after completing each assessment. The study was granted a federal Certificate of Confidentiality, and the data collected were kept confidential."

Your response is too large. Try shortening some answers.

x26-ii) Outline informed consent procedures

Outline informed consent procedures e.g., if consent was obtained offline or online (how? Checkbox, etc.?), and what information was provided (see 4a-ii). See [6] for some items to be included in informed consent documents.

subitem not at all important

1 ○

2 ○

3 ○

4 ○

5 ○

essential

Does your paper address subitem X26-ii?

Copy and paste relevant sections from the manuscript (include quotes in quotation marks "like this" to indicate direct quotes from your manuscript), or elaborate on this item by providing additional information not in the ms, or briefly explain why the item is not applicable/relevant for your study

"All participants provided informed consent before participating in the study."

Your response is too large. Try shortening some answers.

X26-iii) Safety and security procedures

Safety and security procedures, incl. privacy considerations, and any steps taken to reduce the likelihood or detection of harm (e.g., education and training, availability of a hotline)

subitem not at all important

1 ○

2 ○

3 ○

4 ○

5 ○

essential

Does your paper address subitem X26-iii?

Copy and paste relevant sections from the manuscript (include quotes in quotation marks "like this" to indicate direct quotes from your manuscript), or elaborate on this item by providing additional information not in the ms, or briefly explain why the item is not applicable/relevant for your study

"The training sought to support peers in delivering the intervention with fidelity. At the end of the training, the facilitator conducted mock sessions with each of the peers to ensure that they were knowledgeable about all aspects of the intervention and study protocol, covered each of the content areas, provided correct and adequate information, and used appropriate techniques to motivate and empower participants. The PI maintained regular contact and follow-up with peers to support fidelity in the delivery of the intervention. Specifically, the PI supervised all peers, which included regular check-ins with peers over Zoom and email communication regarding the study

RESULTS

Your response is too large. Try shortening some answers.

13a) For each group, the numbers of participants who were randomly assigned, received intended treatment, and were analysed for the primary outcome

NPT: The number of care providers or centers performing the intervention in each group and the number of patients treated by each care provider in each center

Does your paper address CONSORT subitem 13a? *

Copy and paste relevant sections from the manuscript (include quotes in quotation marks "like this" to indicate direct quotes from your manuscript), or elaborate on this item by providing additional information not in the ms, or briefly explain why the item is not applicable/relevant for your study

"We screened 61 individuals for eligibility, 59 (97%) of whom met the eligibility criteria. Of the 59 eligible participants, 50 (85%) agreed to participate. All participants who were eligible and interested in the study provided informed consent. At study completion, 73% (22/30) of the participants in the Listos intervention group and 90% (18/20) of those in the control group completed their 12-week assessment"

13b) For each group, losses and exclusions after randomisation, together with reasons

Does your paper address CONSORT subitem 13b? (NOTE: Preferably, this is shown in a CONSORT flow diagram) *

Copy and paste relevant sections from the manuscript (include quotes in quotation marks "like this" to indicate direct quotes from your manuscript), or elaborate on this item by providing additional information not in the ms, or briefly explain why the item is not applicable/relevant for your study

"We screened 61 individuals for eligibility, 59 (97%) of whom met the eligibility criteria. Of the 59 eligible participants, 50 (85%) agreed to participate. All participants who were eligible and interested in the study provided informed consent. At study completion, 73% (22/30) of the participants in the Listos intervention group and 90% (18/20) of those in the control group completed their 12-week assessment (Figure 1)."

Your response is too large. Try shortening some answers.

13b-i) Attrition diagram

Strongly recommended: An attrition diagram (e.g., proportion of participants still logging in or using the intervention/comparator in each group plotted over time, similar to a survival curve) or other figures or tables demonstrating usage/dose/engagement.

subitem not at all important

1 ○

2 ○

3 ○

4 ○

5 ○

essential

Does your paper address subitem 13b-i?

Copy and paste relevant sections from the manuscript or cite the figure number if applicable (include quotes in quotation marks "like this" to indicate direct quotes from your manuscript), or elaborate on this item by providing additional information not in the ms, or briefly explain why the item is not applicable/relevant for your study

"We screened 61 individuals for eligibility, 59 (97%) of whom met the eligibility criteria. Of the 59 eligible participants, 50 (85%) agreed to participate. All participants who were eligible and interested in the study provided informed consent. At study completion, 73% (22/30) of the participants in the Listos intervention group and 90% (18/20) of those in the control group completed their 12-week assessment (Figure 1)."

14a) Dates defining the periods of recruitment and follow-up

Your response is too large. Try shortening some answers.

Does your paper address CONSORT subitem 14a? *

Copy and paste relevant sections from the manuscript (include quotes in quotation marks "like this" to indicate direct quotes from your manuscript), or elaborate on this item by providing additional information not in the ms, or briefly explain why the item is not applicable/relevant for your study

"Participants were recruited from February 2021 to December 2021 through peers' social networks, word-of-mouth, and referrals."

14a-i) Indicate if critical "secular events" fell into the study period

Indicate if critical "secular events" fell into the study period, e.g., significant changes in Internet resources available or "changes in computer hardware or Internet delivery resources"

subitem not at all important

1 ○

2 ○

3 ○

4 ○

5 ○

essential

Your response is too large. Try shortening some answers.

Does your paper address subitem 14a-i?

Copy and paste relevant sections from the manuscript (include quotes in quotation marks "like this" to indicate direct quotes from your manuscript), or elaborate on this item by providing additional information not in the ms, or briefly explain why the item is not applicable/relevant for your study

"Before peer training in March 2020, the World Health Organization declared COVID-19 a pandemic, and US states began to shut down to prevent the spread of COVID-19 [20]. To protect the safety of peers and to follow local public health guidelines, all research activities were temporarily paused. We subsequently adapted the peer training and intervention procedures to be conducted virtually using internet and phone technologies. The 3-day peer training was conducted over Zoom (Zoom Video Communications), and all relevant materials and training supplies were mailed or

14b) Why the trial ended or was stopped (early)

Does your paper address CONSORT subitem 14b? *

Copy and paste relevant sections from the manuscript (include quotes in quotation marks "like this" to indicate direct quotes from your manuscript), or elaborate on this item by providing additional information not in the ms, or briefly explain why the item is not applicable/relevant for your study

"Before peer training in March 2020, the World Health Organization declared COVID-19 a pandemic, and US states began to shut down to prevent the spread of COVID-19 [20]. To protect the safety of peers and to follow local public health guidelines, all research activities were temporarily paused. We subsequently adapted the peer training and intervention procedures to be conducted virtually using internet and phone technologies. The 3-day peer training was conducted over Zoom (Zoom Video Communications), and all relevant materials and training supplies were mailed or

15) A table showing baseline demographic and clinical characteristics for each group

NPT: When applicable, a description of care providers (case volume, qualification, expertise, etc.) and centers (volume) in each group

Your response is too large. Try shortening some answers.

Does your paper address CONSORT subitem 15? *

Copy and paste relevant sections from the manuscript (include quotes in quotation marks "like this" to indicate direct quotes from your manuscript), or elaborate on this item by providing additional information not in the ms, or briefly explain why the item is not applicable/relevant for your study

"Table 2 presents the baseline demographic characteristics of participants in the intervention and control groups."

15-i) Report demographics associated with digital divide issues

In ehealth trials it is particularly important to report demographics associated with digital divide issues, such as age, education, gender, social-economic status, computer/Internet/ehealth literacy of the participants, if known.

subitem not at all important

1 ○

2 ○

3 ○

4 ○

5 ○

essential

Your response is too large. Try shortening some answers.

Does your paper address subitem 15-i? *

Copy and paste relevant sections from the manuscript (include quotes in quotation marks "like this" to indicate direct quotes from your manuscript), or elaborate on this item by providing additional information not in the ms, or briefly explain why the item is not applicable/relevant for your study

"Most participants (41/50, 82%) completed the survey in Spanish, and the overall mean age was 37.3 years. Overall, 62% (31/50) of participants reported being born in Mexico, and less than one-third of the participants (15/50, 30%) indicated having ever returned to their country of origin since migrating to the United States. Approximately a quarter of the participants (13/50, 26%) indicated that they were legal permanent residents, and another quarter (12/50, 24%) indicated that they were unauthorized immigrants. Moreover, 70% (35/50) of participants were identified as Christian or Catholic. Less than half of participants (22/50, 44%) had medical insurance. Sociodemographic characteristics did not differ significantly between the groups."

16) For each group, number of participants (denominator) included in each analysis and whether the analysis was by original assigned groups

16-i) Report multiple “denominators” and provide definitions

Report multiple “denominators” and provide definitions: Report N’s (and effect sizes) “across a range of study participation [and use] thresholds” [1], e.g., N exposed, N consented, N used more than x times, N used more than y weeks, N participants “used” the intervention/comparator at specific pre-defined time points of interest (in absolute and relative numbers per group). Always clearly define “use” of the intervention.

subitem not at all important

1 ○

2 ○

3 ○

4 ○

5 ○

Your response is too large. Try shortening some answers.

Does your paper address subitem 16-i? *

Copy and paste relevant sections from the manuscript (include quotes in quotation marks "like this" to indicate direct quotes from your manuscript), or elaborate on this item by providing additional information not in the ms, or briefly explain why the item is not applicable/relevant for your study

"With respect to HIV testing results, the vast majority of participants (48/50, 96%) reported having ever being tested for HIV, and approximately one-third of the participants (18/50, 36%) reported having ever used PrEP at baseline. Differences in HIV testing, STI testing, and PrEP uptake outcomes between the intervention and control groups after participation in the program are presented in Table 4. Most participants indicated that the program motivated them to get tested for HIV and STIs (proportion in the intervention group: 23/25, 92.0% and 24/25, 96.0%, respectively; proportion in the control group: 12/18, 66.7% and 13/19, 68.4%, respectively; P<.05). The analysis revealed a significant association between assigned group and motivation to get tested for HIV (Cramer V=0.321; P=.04) and motivation to get tested for STIs (Cramer V=0.373; P=.01). A considerable proportion of all participants (30/44, 68.2%) reported testing for HIV after participating in the program. There were no differences in the reports of HIV testing after completion of the program between the intervention and control groups. A greater proportion of participants in the intervention group reported testing for STIs than those in the control group (19/25, 76.0% vs 7/19, 36.8%; P=.009; Cramer V=0.394). The overall proportion of participants who indicated that the program motivated them to use PrEP was 77.5% (31/40), with 91.3% (21/23) in the intervention group and 58.8% (10/17) in the control group (Cramer V=0.385; P=.02). Overall, of the 43 participants, 10 (23.3%) reported initiating PrEP after participating in the program (n=8, 80% in the intervention group and n=2, 20% in the control group)."

Your response is too large. Try shortening some answers.

16-ii) Primary analysis should be intent-to-treat

Primary analysis should be intent-to-treat, secondary analyses could include comparing only “users”, with the appropriate caveats that this is no longer a randomized sample (see 18-i).

subitem not at all important

1 ○

2 ○

3 ○

4 ○

5 ○

essential

Does your paper address subitem 16-ii?

Copy and paste relevant sections from the manuscript (include quotes in quotation marks "like this" to indicate direct quotes from your manuscript), or elaborate on this item by providing additional information not in the ms, or briefly explain why the item is not applicable/relevant for your study

"Among the 30 Listos intervention group participants who received the HIV and STI self-testing kits, 20 (67%) completed kits that were received by the testing laboratory. Notably, only 19 intervention group participants self-reported having been tested for HIV after participating in the program (Table 4), which may have resulted from differences in the timing of assessment completion and completion of the testing kit. Five of these participants had positive results: 4 for HSV-2, 2 for syphilis, and 1 for HIV. For 3 participants, positive results for HSV-2 were new diagnoses based on self-reports. All 5 participants who received a positive test result received counseling from their peers and were linked to appropriate follow-up testing and care through local community–based service providers."

17a) For each primary and secondary outcome, results for each group, and the [illegible]

Your response is too large. Try shortening some answers.

Does your paper address CONSORT subitem 17a? *

Copy and paste relevant sections from the manuscript (include quotes in quotation marks "like this" to indicate direct quotes from your manuscript), or elaborate on this item by providing additional information not in the ms, or briefly explain why the item is not applicable/relevant for your study

"With respect to HIV testing results, the vast majority of participants (48/50, 96%) reported having ever being tested for HIV, and approximately one-third of the participants (18/50, 36%) reported having ever used PrEP at baseline. Differences in HIV testing, STI testing, and PrEP uptake outcomes between the intervention and control groups after participation in the program are presented in Table 4. Most participants indicated that the program motivated them to get tested for HIV and STIs (proportion in the intervention group: 23/25, 92.0% and 24/25, 96.0%, respectively; proportion in the control group: 12/18, 66.7% and 13/19, 68.4%, respectively; P<.05). The analysis revealed a significant association between assigned group and motivation to get tested for HIV (Cramer V=0.321; P=.04) and motivation to get tested for STIs (Cramer V=0.373; P=.01). A considerable proportion of all participants (30/44, 68.2%) reported testing for HIV after participating in the program. There were no differences in the reports of HIV testing after completion of the program between the intervention and control groups. A greater proportion of participants in the intervention group reported testing for STIs than those in the control group (19/25, 76.0% vs 7/19, 36.8%; P=.009; Cramer V=0.394). The overall proportion of participants who indicated that the program motivated them to use PrEP was 77.5% (31/40), with 91.3% (21/23) in the intervention group and 58.8% (10/17) in the control group (Cramer V=0.385; P=.02). Overall, of the 43 participants, 10 (23.3%) reported initiating PrEP after participating in the program (n=8, 80% in the intervention group and n=2, 20% in the control group)."

Your response is too large. Try shortening some answers.

17a-i) Presentation of process outcomes such as metrics of use and intensity of use

In addition to primary/secondary (clinical) outcomes, the presentation of process outcomes such as metrics of use and intensity of use (dose, exposure) and their operational definitions is critical. This does not only refer to metrics of attrition (13-b) (often a binary variable), but also to more continuous exposure metrics such as “average session length”. These must be accompanied by a technical description how a metric like a “session” is defined (e.g., timeout after idle time) [1] (report under item 6a).

subitem not at all important

1 ○

2 ○

3 ○

4 ○

5 ○

essential

Does your paper address subitem 17a-i?

Copy and paste relevant sections from the manuscript (include quotes in quotation marks "like this" to indicate direct quotes from your manuscript), or elaborate on this item by providing additional information not in the ms, or briefly explain why the item is not applicable/relevant for your study

N/A- Our study did not evaluate metrics or intensity of use.

17b) For binary outcomes, presentation of both absolute and relative effect sizes is recommended

Your response is too large. Try shortening some answers.

Does your paper address CONSORT subitem 17b? *

Copy and paste relevant sections from the manuscript (include quotes in quotation marks "like this" to indicate direct quotes from your manuscript), or elaborate on this item by providing additional information not in the ms, or briefly explain why the item is not applicable/relevant for your study

With respect to HIV testing results, the vast majority of participants (48/50, 96%) reported having ever being tested for HIV, and approximately one-third of the participants (18/50, 36%) reported having ever used PrEP at baseline. Differences in HIV testing, STI testing, and PrEP uptake outcomes between the intervention and control groups after participation in the program are presented in Table 4. Most participants indicated that the program motivated them to get tested for HIV and STIs (proportion in the intervention group: 23/25, 92.0% and 24/25, 96.0%, respectively; proportion in the control group: 12/18, 66.7% and 13/19, 68.4%, respectively; $P<.05$). The analysis revealed a significant association between assigned group and motivation to get tested for HIV (Cramer V=0.321; $P=.04$) and motivation to get tested for STIs

18) Results of any other analyses performed, including subgroup analyses and adjusted analyses, distinguishing pre-specified from exploratory

Does your paper address CONSORT subitem 18? *

Copy and paste relevant sections from the manuscript (include quotes in quotation marks "like this" to indicate direct quotes from your manuscript), or elaborate on this item by providing additional information not in the ms, or briefly explain why the item is not applicable/relevant for your study

"Table 3 presents the characteristics related to COVID-19 among the participants overall and by intervention and control groups. There were no differences between the arms. At baseline, approximately one-third of participants reported having experienced COVID-19 symptoms, whereas the vast majority of participants indicated having been tested for COVID-19. Overall, 28% (12/43) of participants who had tested for COVID-19 had received a positive test result, and more than two-thirds of the participants (34/50, 68%) had lost their jobs since the declaration of the COVID-19 pandemic. The average impact of COVID-19 on participants' lives was 3.4 on a scale of 1 to 5, and the average level of distress experienced in relation to COVID-19 was 5.9 on a scale of 1 to 10. Approximately half of the participants (26/50, 52%) noted that they were scared of

Your response is too large. Try shortening some answers.

18-i) Subgroup analysis of comparing only users

A subgroup analysis of comparing only users is not uncommon in ehealth trials, but if done, it must be stressed that this is a self-selected sample and no longer an unbiased sample from a randomized trial (see 16-iii).

subitem not at all important

1 ○

2 ○

3 ○

4 ○

5 ○

essential

Does your paper address subitem 18-i?

Copy and paste relevant sections from the manuscript (include quotes in quotation marks "like this" to indicate direct quotes from your manuscript), or elaborate on this item by providing additional information not in the ms, or briefly explain why the item is not applicable/relevant for your study

N/A- Our paper does not conduct subgroup analysis of comparing only users.

19) All important harms or unintended effects in each group

(for specific guidance see CONSORT for harms)

Your response is too large. Try shortening some answers.

Does your paper address CONSORT subitem 19? *

Copy and paste relevant sections from the manuscript (include quotes in quotation marks "like this" to indicate direct quotes from your manuscript), or elaborate on this item by providing additional information not in the ms, or briefly explain why the item is not applicable/relevant for your study

"The implementation of our peer-based intervention during the global pandemic was novel. As indicated by our results, Latinx SMM were greatly affected by the COVID-19 pandemic, with a significant number reporting job loss and distress. The social and economic effects of COVID-19 continue to be documented, demonstrating disproportionate impacts on immigrant communities in the United States [27]. Furthermore, HIV testing and screening for STIs were disrupted during the COVID-19 pandemic, with data pointing to substantial declines in HIV and STI testing rates from 2019 to 2020 [28-30]."

19-i) Include privacy breaches, technical problems

Include privacy breaches, technical problems. This does not only include physical “harm” to participants, but also incidents such as perceived or real privacy breaches [1], technical problems, and other unexpected/unintended incidents. “Unintended effects” also includes unintended positive effects [2].

subitem not at all important

1 ○

2 ○

3 ○

4 ○

5 ○

essential

Your response is too large. Try shortening some answers.

Does your paper address subitem 19-i?

Copy and paste relevant sections from the manuscript (include quotes in quotation marks "like this" to indicate direct quotes from your manuscript), or elaborate on this item by providing additional information not in the ms, or briefly explain why the item is not applicable/relevant for your study

N/A- there were no privacy breaches or technical problems to report.

19-ii) Include qualitative feedback from participants or observations from staff/researchers

Include qualitative feedback from participants or observations from staff/researchers, if available, on strengths and shortcomings of the application, especially if they point to unintended/unexpected effects or uses. This includes (if available) reasons for why people did or did not use the application as intended by the developers.

subitem not at all important

1 ○

2 ○

3 ○

4 ○

5 ○

essential

Does your paper address subitem 19-ii?

Copy and paste relevant sections from the manuscript (include quotes in quotation marks "like this" to indicate direct quotes from your manuscript), or elaborate on this item by providing additional information not in the ms, or briefly explain why the item is not applicable/relevant for your study

N/A- we do not report on qualitative feedback from participants or observations from [illegible]

Your response is too large. Try shortening some answers.

DISCUSSION

22) Interpretation consistent with results, balancing benefits and harms, and considering other relevant evidence

NPT: In addition, take into account the choice of the comparator, lack of or partial blinding, and unequal expertise of care providers or centers in each group

22-i) Restate study questions and summarize the answers suggested by the data, starting with primary outcomes and process outcomes (use)

Restate study questions and summarize the answers suggested by the data, starting with primary outcomes and process outcomes (use).

subitem not at all important

1 ○

2 ○

3 ○

4 ○

5 ○

essential

Your response is too large. Try shortening some answers.

Does your paper address subitem 22-i? *

Copy and paste relevant sections from the manuscript (include quotes in quotation marks "like this" to indicate direct quotes from your manuscript), or elaborate on this item by providing additional information not in the ms, or briefly explain why the item is not applicable/relevant for your study

"This study demonstrated the feasibility of the Listos program, a peer-based HIV and STI intervention conducted virtually during the COVID-19 pandemic. The goal of the program was to promote HIV testing, STI testing, and PrEP use among Latinx immigrant SMM in Washington State. Our primary objective was to examine the differences in HIV testing, STI testing, and PrEP use behaviors between the intervention and control groups. In addition, given our program's implementation during the COVID-19 pandemic, we sought to assess the impact of the pandemic on the program participants."

22-ii) Highlight unanswered new questions, suggest future research

Highlight unanswered new questions, suggest future research.

subitem not at all important

1 ○

2 ○

3 ○

4 ○

5 ○

essential

Your response is too large. Try shortening some answers.

Does your paper address subitem 22-ii?

Copy and paste relevant sections from the manuscript (include quotes in quotation marks "like this" to indicate direct quotes from your manuscript), or elaborate on this item by providing additional information not in the ms, or briefly explain why the item is not applicable/relevant for your study

"Future studies with long-term evaluations are needed to assess the costs and sustainability of such programs. In addition, future research may explore opportunities to integrate web-based technology to support and develop peer-based interventions to enhance HIV prevention and care for Latinx and immigrant communities."

20) Trial limitations, addressing sources of potential bias, imprecision, and, if relevant, multiplicity of analyses

20-i) Typical limitations in ehealth trials

Typical limitations in ehealth trials: Participants in ehealth trials are rarely blinded. Ehealth trials often look at a multiplicity of outcomes, increasing risk for a Type I error. Discuss biases due to non-use of the intervention/usability issues, biases through informed consent procedures, unexpected events.

subitem not at all important

1 ○

2 ○

3 ○

4 ○

5 ○

essential

Your response is too large. Try shortening some answers.

Does your paper address subitem 20-i? *

Copy and paste relevant sections from the manuscript (include quotes in quotation marks "like this" to indicate direct quotes from your manuscript), or elaborate on this item by providing additional information not in the ms, or briefly explain why the item is not applicable/relevant for your study

"This study has several limitations. First, participants were recruited by peers through their social networks and through web-based strategies using convenience sampling. Hence, the results cannot be generalized to all Latinx immigrant SMM. Peers may have known some of the participants previously, which may have biased the results. Second, because of challenges related to the COVID-19 pandemic, there was greater loss to follow-up than anticipated. Third, the small sample size limited our statistical analyses, which consequently could not be controlled for factors that differed across study arms. Therefore, our analyses require further confirmation. Fourth, because the peer counseling component was delivered to both arms, we could not determine whether this component of the intervention had any impact on outcomes. In addition, we cannot attribute the uptake of self-testing kits to peers, given that both groups were exposed to peer counseling. Finally, the intervention was delivered in 2021, and the effects of the COVID-19 pandemic on access to health services should be considered when interpreting the results."

21) Generalisability (external validity, applicability) of the trial findings

NPT: External validity of the trial findings according to the intervention, comparators, patients, and care providers or centers involved in the trial

Your response is too large. Try shortening some answers.

21-i) Generalizability to other populations

Generalizability to other populations: In particular, discuss generalizability to a general Internet population, outside of a RCT setting, and general patient population, including applicability of the study results for other organizations

subitem not at all important

1 ○

2 ○

3 ○

4 ○

5 ○

essential

Does your paper address subitem 21-i?

Copy and paste relevant sections from the manuscript (include quotes in quotation marks "like this" to indicate direct quotes from your manuscript), or elaborate on this item by providing additional information not in the ms, or briefly explain why the item is not applicable/relevant for your study

"First, participants were recruited by peers through their social networks and through web-based strategies using convenience sampling. Hence, the results cannot be generalized to all Latinx immigrant SMM."

Your response is too large. Try shortening some answers.

21-ii) Discuss if there were elements in the RCT that would be different in a routine application setting

Discuss if there were elements in the RCT that would be different in a routine application setting (e.g., prompts/reminders, more human involvement, training sessions or other co-interventions) and what impact the omission of these elements could have on use, adoption, or outcomes if the intervention is applied outside of a RCT setting.

subitem not at all important

1 ○

2 ○

3 ○

4 ○

5 ○

essential

Does your paper address subitem 21-ii?

Copy and paste relevant sections from the manuscript (include quotes in quotation marks "like this" to indicate direct quotes from your manuscript), or elaborate on this item by providing additional information not in the ms, or briefly explain why the item is not applicable/relevant for your study

"For our participants, self-collection STI testing using a free kit delivered directly to their homes might have been a vital means of overcoming access barriers. Although these results support our intervention, the costs of the kits and the feasibility of kit delivery and their use outside of a research study should be considered."

OTHER INFORMATION

23) Registration number and name of trial registry

Your response is too large. Try shortening some answers.

Does your paper address CONSORT subitem 23? *

Copy and paste relevant sections from the manuscript (include quotes in quotation marks "like this" to indicate direct quotes from your manuscript), or elaborate on this item by providing additional information not in the ms, or briefly explain why the item is not applicable/relevant for your study

"Trial Registration: ClinicalTrials.gov NCT03922126"

24) Where the full trial protocol can be accessed, if available

Does your paper address CONSORT subitem 24? *

Cite a Multimedia Appendix, other reference, or copy and paste relevant sections from the manuscript (include quotes in quotation marks "like this" to indicate direct quotes from your manuscript), or elaborate on this item by providing additional information not in the ms, or briefly explain why the item is not applicable/relevant for your study

"https://clinicaltrials.gov/ct2/show/NCT03922126"

25) Sources of funding and other support (such as supply of drugs), role of funders

Does your paper address CONSORT subitem 25? *

Copy and paste relevant sections from the manuscript (include quotes in quotation marks "like this" to indicate direct quotes from your manuscript), or elaborate on this item by providing additional information not in the ms, or briefly explain why the item is not applicable/relevant for your study

"This project was supported by the National Center For Advancing Translational Sciences of the National Institutes of Health (KL2TR000421). The content is solely the responsibility of the authors and does not necessarily represent the official views of the National Institutes of Health."

Your response is too large. Try shortening some answers.

X27) Conflicts of Interest (not a CONSORT item)

X27-i) State the relation of the study team towards the system being evaluated

In addition to the usual declaration of interests (financial or otherwise), also state the relation of the study team towards the system being evaluated, i.e., state if the authors/evaluators are distinct from or identical with the developers/sponsors of the intervention.

subitem not at all important

1 ○

2 ○

3 ○

4 ○

5 ○

essential

Does your paper address subitem X27-i?

Copy and paste relevant sections from the manuscript (include quotes in quotation marks "like this" to indicate direct quotes from your manuscript), or elaborate on this item by providing additional information not in the ms, or briefly explain why the item is not applicable/relevant for your study

"JCD has conducted research with donations from Hologic and Mayne

About the CONSORT EHEALTH checklist

Your response is too large. Try shortening some answers.

As a result of using this checklist, did you make changes in your manuscript? *

- ○ yes, major changes
- ◉ yes, minor changes
- ○ no

What were the most important changes you made as a result of using this checklist?

Your answer

How much time did you spend on going through the checklist INCLUDING making changes in your manuscript *

I spent approximately two hours going through the checklist.

As a result of using this checklist, do you think your manuscript has improved? *

- ◉ yes
- ○ no
- ○ Other:

Your response is too large. Try shortening some answers.

Would you like to become involved in the CONSORT EHEALTH group?

This would involve for example becoming involved in participating in a workshop and writing an "Explanation and Elaboration" document

- ○ yes
- ○ no
- ◉ Other:

Clear selection

Any other comments or questions on CONSORT EHEALTH

Your answer

STOP - Save this form as PDF before you click submit

To generate a record that you filled in this form, we recommend to generate a PDF of this page (on a Mac, simply select "print" and then select "print as PDF") before you submit it.

When you submit your (revised) paper to JMIR, please upload the PDF as supplementary file.

Don't worry if some text in the textboxes is cut off, as we still have the complete information in our database. Thank you!

Final step: Click submit !

Click submit so we have your answers in our database!

Submit Clear form

Never submit passwords through Google Forms.

This content is neither created nor endorsed by Google. Report Abuse - Terms of Service - Privacy Policy

Your response is too large. Try shortening some answers.

Your response is too large. Try shortening some answers.
